# Causes of Death after Prostate Cancer Diagnosis: A Population-Based Study

**DOI:** 10.1155/2022/8145173

**Published:** 2022-04-23

**Authors:** Yadong Guo, Xiaohui Dong, Shiyu Mao, Fuhan Yang, Ruiliang Wang, Wenchao Ma, Ji Liu, Cheng Li, Zongtai Zheng, Wentao Zhang, Aihong Zhang, Xudong Yao

**Affiliations:** ^1^Department of Urology, Shanghai Tenth People's Hospital, School of Medicine, Tongji University, Shanghai 200072, China; ^2^Department of Special Medical, Shanghai Fourth People's Hospital, School of Medicine, Tongji University, Shanghai 200434, China; ^3^Department of Medical Statistics, Tongji University School of Medicine, Shanghai 200092, China

## Abstract

**Background:**

Mortality from noncancer causes in patients with prostate cancer (PCa) is unclear. This study assesses the causes and risks of noncancer death with each follow-up time period after PCa diagnosis.

**Methods:**

Data from the Surveillance, Epidemiology, and End Results (SEER) program were analyzed for noncancer causes of death in PCa patients from 2000 to 2016. The standard mortality ratio (SMR) was calculated for noncancer mortality.

**Results:**

Altogether, 752,352 patients with PCa were identified, and 180,862 (24.0%) died during follow-up. The largest proportion of deaths from noncancer causes (36%) occurred within 5 to 10 years after diagnosis. The most common causes of noncancer death are cardiovascular and cerebrovascular diseases and chronic obstructive pulmonary disease (COPD). Compared with the general age-matched male population, patients with PCa had a higher risk of death from any noncancer cause within 5 years, in particular other infectious diseases and suicide and self-inflicted injury. However, the risk of death from noncancer causes of PCa for more than 5 years is lower, except for Alzheimer's disease and hypertension from 5 to 10 years after diagnosis. In addition, the risk of death from noncancer causes was influenced by treatment, ethnicity, and staging differences. In particular, compared with the general population, many noncancer causes of death have higher risk of death in patients with or without treatment within 1 to 5 years after diagnosis, whereas patients undergoing radical prostatectomy (RP) with or without radiotherapy (RT) or chemotherapy (CTx) are not at high risk of death from COPD, pneumonia and influenza, nephritis, nephrotic syndrome and nephrosis, septicemia, and atherosclerosis.

**Conclusion:**

The risk of death from noncancer causes gradually decreased in all patients with PCa during each follow-up period after diagnosis In addition, the risk of dying from noncancer causes are influenced by differences in stage, ethnicity, and treatment. In particular, patients undergoing RP±RT/CTx and RT/CTx have a lower risk of death compared to the general population. These findings provide important implications for the healthcare management of patients with PCa.

## 1. Introduction

Prostate cancer (PCa) is the most common primary malignancy and the second leading cause of cancer death among men in the United States [1]. An estimated 248,530 Americans were diagnosed with PCa in 2021, of which 34,130 died [1]. However, due to improvements in screening and treatment, early cancer diagnosis is associated with reduced cancer mortality. Therefore, since its peak in 1992, the survival rate of PCa has shown an upward trend [2, 3].

In the past few decades, patients with PCa have often survived long enough to die of other causes, a circumstance that calls into question the influence of PCa on survival [4–6]. Previous studies investigating the cause of death in patients with PCa found that cardiovascular disease was paramount [7, 8]. Indeed, according to some studies with long-term follow-ups of patients with local PCa, the risk of PCa-specific mortality is significantly lower than the risk of death from other causes [9, 10]. In general, these studies show that the causes of death vary widely according to the different clinical characteristics of patients. These results may not reflect the impact of improved PCa-based screening and treatment on the prognosis of PCa.

Given the long-term epidemiological trends in PCa, our study established an association between causes of death and demographics in PCa and provided results for the stratification of patient characteristics. Finally, we compared the risk of each cause of death with that of the general population in the United States over the same period.

## 2. Methods

### 2.1. Database and Patient Selection

The SEER program collects data from the National Cancer Registry, which covers approximately 34.6% of the population. Since 1999, the SEER registry has recorded the cause of death based on the International Classification of Diseases (ICD-10). Table S1 includes definitions of each cause of death by the ICD-10 code.

For this study, we considered all patients with first primary PCa that were diagnosed between the years 2000 and 2016. The following variables were analyzed: marital status (married, widowed or divorced (W/D), single, and unknown); ethnicity (White, Black, other, and unknown); age at diagnosis (<50, 50-64, 65-74, or ≥75 years); year of diagnosis (in the years 2000-2005 or 2006-2016); stage (local, regional, and distant) and grade (I-IV and unknown); therapy type (none, radical prostatectomy (RP), and radiotherapy (RT)/chemotherapy (CTx)); survival time (<1, 1-5, 5-10, or >10 years); and detailed causes of death.

### 2.2. Statistical Analysis

SEER∗Stat statistical software (version 8.3.6) was used to calculate the frequency of categorical variables and the percentage of deaths due to each clinicopathological variable. And the distribution of each cause of death was summarized for each survival time category. The Fine-Gray competitive risk model was applied to adjust for confounding effects of age at diagnosis, year of diagnosis, race, marital status, tumor stage, grade, and treatment, to evaluate risks for other CODs and PCSM, and to plot a crude cumulative mortality curve.

To investigate whether risk factors such as age were associated with each noncancer cause of death, the standardized-mortality ratios (SMRs) were calculated for each cause of death post PCa diagnosis. Mortality for the general population was selected from the Centers for Disease Control and Prevention (CDC) WONDER Mortality Underlying Cause of Death online database [11]. The SMRs for a specific cause after PCa diagnosis are the ratio of the total number of observed deaths to the number expected from age-specific reference rates. The expected numbers were calculated by multiplying the cumulative person-time across patients with PCa within 5-year age groups (35–39, 40–44, 50–54, 55–59, 60–64, 65–69, 70–74, 75–79, 80–84, and >85 years) and the calendar period 2000–2016 with the age-, sex-, and race- and calendar period-specific mortality from a specific cause of death in the general population. The SMRs were calculated with a 95% confidence interval, and a *P* value < 0.05 was considered statistically significant. The calculation of SMR and *P* values for groups with less than 10 males was deleted. All statistical tests were two-sided. All analyses were performed using Stata/MP 14.0 and R software packages.

## 3. Results

### 3.1. Patient Characteristics

There were 752,352 patients with PCa identified in the SEER database from 2000 to 2016. The age group with the largest proportion was 50-64 years of age (302301, 40.2%). The majority were married (484508, 64.4%), White (576407, 76.6%), and with local PCa (587431, 78.1%). The largest tumor grade group was grade II (357337, 47.5%). The median survival time for the overall population was 95 months (95% CI: 94.7-95.2 months).

Table 1 lists the clinicopathological characteristics of patients with different death times and proportion of death after diagnosis of PCa. During follow-up, 180,862 (24.0%) of the men died, with an average age at death of 73.3 years. The highest number of deaths (70,001; 38.7%) occurred 1 to 5 years post PCa was diagnosed (Table 1). Moreover, 25,004 (13.8%) deaths occurred in ≤1 year, 57,150 (31.6%) within 5 to 10 years, and 28,707 (15.9%) at >10 years after the PCa diagnosis.

### 3.2. Noncancer Causes of Death at Different Time Intervals Postdiagnosis

#### 3.2.1. Within 5 Years after PCa Diagnosis

**Figure 1:**
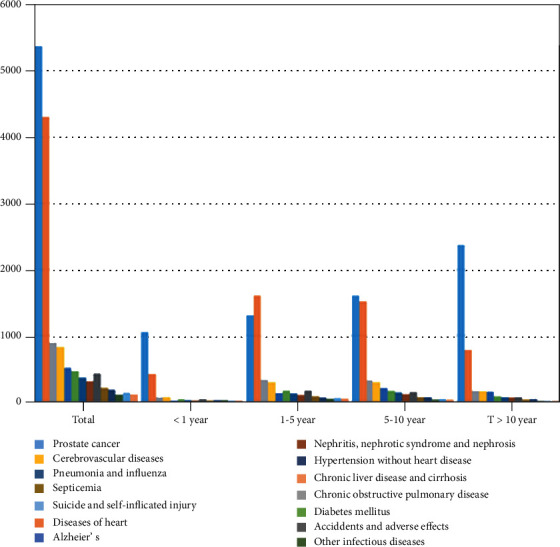
Causes of death listed following a diagnosis of prostate cancer, stratified by the year after diagnosis.

Within the first year after diagnosis, 25,004 (3.3%) of the overall population (*n* = 752,352) died (Table 2 and Figure 1). Among these, 10,507 (42.0%) died from PCa; 692 (2.8%) died from other cancers; and 13805 (55.2%) died from noncancer causes. Within 1 to 5 years after diagnosis, 70001 (9.3%) of the overall population (*n* = 752,352) died (Table 2 and Figure 1). Among these, 26,014 (37.2%) died from PCa; 2012 (2.8%) died from other cancers; and 41975 (60%) died from noncancer causes. The most common noncancer causes of death are heart disease, cerebrovascular disease, and chronic obstructive pulmonary disease (COPD) in the first year after PCa diagnosis. However, from 1 to 5 years after PCa diagnosis, it was followed by heart disease, COPD, and cerebrovascular disease.

**Figure 2:**
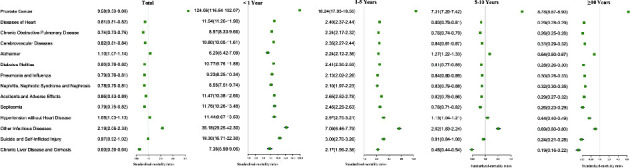
SMR by cause of death, stratified by year after diagnosis.

The risk of dying from noncancer causes within 5 years after PCa diagnosis was significantly higher than that of the general population, especially other infectious diseases and suicide and self-inflicted injury (Table 2 and Figure 2).

#### 3.2.2. More than 5 Years after PCa Diagnosis

Within 5 to 10 years after diagnosis, 57,150 (7.6%) of the overall population (*n* = 752,352) died (Table 2 and Figure 1). Among these, 12,670 (22.2%) died from PCa; 1,879 (3.3%) died from other cancers; and 42,601 (74.5%) died from noncancer causes. Approximately 28,707 (3.8%) of the study population died >10 years after the PCa diagnosis (Table 2 and Figure 1). Among them, 4,537 (15.8%) died of PCa, 1041 (3.6%) died of other cancers, and 23,129 (80.6%) died of noncancer causes. The most common noncancer causes of death more than 5 years after PCa diagnosis were heart disease, COPD, and cerebrovascular disease. Compared with the general population, men from 5 to 10 years after PCa diagnosis were at significantly higher risk of death from other infectious diseases, Alzheimer's disease, and hypertension, while that of more than 10 years were at significantly lower risk of death from all noncancer causes (Table 2 and Figure 2).

#### 3.2.3. Noncancer Causes of Death according to Different Subgroups

According to the clinicopathological characteristics, the causes of noncancer deaths with each follow-up time period after PCa diagnosis were further analyzed by each specific subgroup (Table S2-23). In addition, some meaningful results are as follows:

#### 3.2.4. Tumor Stage

Among them, 122536 (21.0%) died from locally diagnosed PCa, 14923 (16.3%) died from regionally diagnosed PCa, and 26426 (71.9%) died of PCa diagnosed with distant-stage. The highest proportion of deaths of patients with localized PCa occurred 5-10 years after diagnosis, and that of regional and distant-stage PCa occurred 1-5 years after diagnosis, especially noncancer causes of death (Table S12-14). In addition, compared with the general population, patients with localized PCa has a higher risk of death from Alzheimer's disease, hypertension, and other infections; patients with regional PCa have a higher risk of death from Alzheimer's disease and other infections; patients with distant PCa have a lower risk of death from Alzheimer's disease, chronic liver disease, and aortic dissection. Furthermore, patients with regional PCa have a significantly higher risk of dying from other infectious diseases and hypertension without heart disease within the first year of diagnosis. However, patients with distant-stage PCa have no higher risk of dying from heart disease and Alzheimer's within 1 to 5 years after diagnosis, while that of more than 5 years were at significantly lower risk of death from all noncancer causes (Table S12-14).

#### 3.2.5. Ethnicity

139,025 (24.6%) PCa patients who died were White, 28,545 (25.6%) were Black, and 8,690 (24.8%) were other races. The highest proportion of deaths occurred 1-5 years after diagnosis that included noncancer causes of death (Table S9-11). Furthermore, compared with the general population, White patients had a higher risk of death from Alzheimer's disease, suicide, and other infectious diseases, whereas Black patients had not a higher risk of death from COPD, suicide, chronic liver disease, accidents, and aortic dissection; patients of other races have a higher risk of death from pneumonia. In addition, Black patients were at higher risk of other infectious diseases and septicemia than the general population within the first year after the cancer diagnosis, and the risk of death of other races of patients that suffered from pneumonia and influenza and nephritis, nephrotic syndrome, and nephrosis is significantly higher within 1 to 5 years after a cancer diagnosis. In addition, within 5 to 10 years after PCa diagnosis, Black patients were at higher risk of heart disease, cerebrovascular disease, diabetes, kidney disease, and septicemia than the general population (Table S9-11).

#### 3.2.6. Treatment

Approximately 94,581 (38.6%) PCa patients who did not receive any treatment for PCa died, 18,532 (7.6%) who underwent RP±RT/CTx died, and 59,788 (24.8%) who underwent RT/CTx died (Table S21-23). The highest proportion of deaths of patients with no treatment occurred 1-5 years after diagnosis, and that of received treatment PCa occurred 5-10 years after diagnosis, especially noncancer causes of death. In addition, PCa patients with no treatment were at significantly higher risk of death from all noncancer causes except for chronic liver disease and aortic aneurysms and dissections, compared with the general population. The risk of noncancer causes of death for patients undergoing RP±RT/CTx and RT/CTx is lower than that of the general population. Furthermore, compared with the general population, many noncancer causes of death have higher risk of death in patients with or without treatment within 1 to 5 years after diagnosis, whereas patients undergoing RP±RT/CTx are not at high risk of death from COPD, pneumonia and influenza, nephritis, nephrotic syndrome and nephrosis, septicemia, and atherosclerosis (Table S21-23).

Interestingly, in patients with 65+ years, the risk of death from Alzheimer's disease is higher than that of the general population, as well during the 1-10 years following diagnosis (Table S4-5). Furthermore, compared with the general population, grade I patients from 5 to 10 years had higher risk, with the exception of diabetes mellitus, nephritis, nephrotic syndrome and nephrosis, accidents and adverse effects, and septicemia, whereas Alzheimer's in patients with grade III has a low risk of death (Table S15, 17). However, patients had a significantly higher risk of death from all noncancer causes compared with the general population, if the PCa diagnosis occurred during the years 2000-2005. Conversely, the risk of death for patients given the PCa diagnosis during the years 2006 to 2016 was significantly lower (Table S19-20).

### 3.3. Relative Risk Model for Other CODs and PCSM

**Figure 3:**
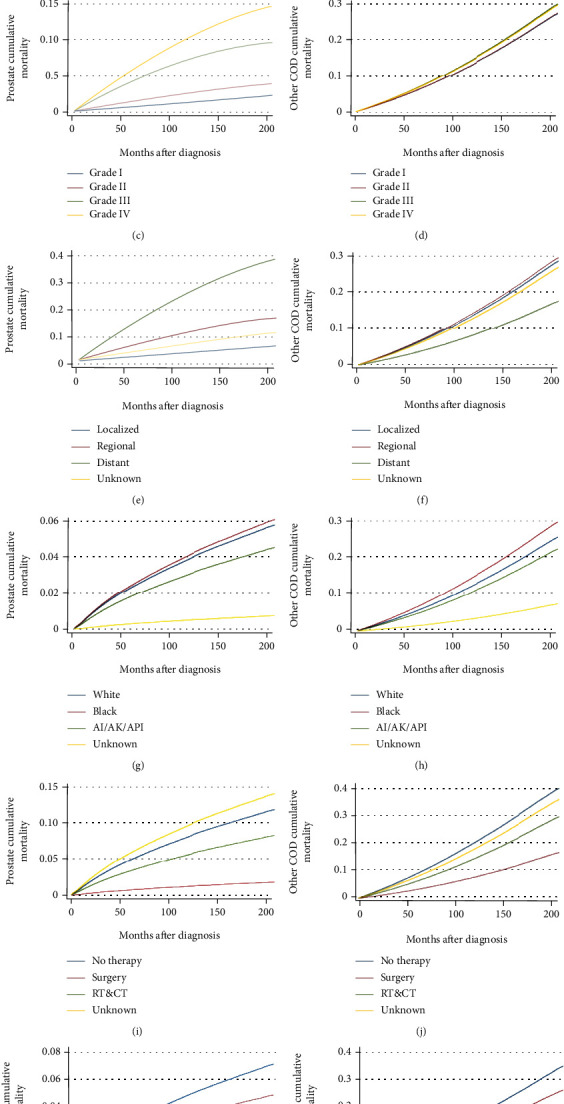
Multivariable adjusted cumulative incidence function curve of patients with prostate cancer (PCa). Probability of PCa-specific mortality (PCSM) and other causes of death (CODs) by (a and b) age; (c and d) tumor grade; (e and f) stage; (g and h) race; (i and j) therapy; and (k and l) year of diagnosis.

The Fine-Gray model of competitive risk was used to assess other prognostic factors and cumulative mortality for COD or PCSM in patients with PCa (Table 3 and Figure 3). The risk of other CODs and PCSM increased with age at diagnosis of PCa or with increased follow-up time (Figures 3(a) and 3(b)). In particular, the risk for other COD increased gradually from 1.97 (from 50 years of age) to 10.51 (over 75 years of age) with each 5-year increase in age (Table 3). In addition, the risk of PCSM increased significantly with the increase in the grade of PCa diagnosed, while there was no difference in other COD risks between grade I and grades II and IV (Figures 3(c) and 3(d) and Table 3). Compared with patients with local disease, those with regional showed a significantly higher risk of other CODs and PCSM. However, the risk of other CODs in patients with distant disease was significantly less relative to local disease (Figures 3(e) and 3(f) and Table 3). Compared with White patients, Black patients had a higher risk of other CODs and PCSM, while patients of other ethnicities had a lower risk (Figures 3(g) and 3(h) and Table 3). Widowed/divorced and single patients had a higher risk of other COD and PCSM than married patients (Table 3). In addition, the risk of other COD and PCSM was significantly lower in patients receiving RP±RT/CT and RT/CT than in patients not receiving treatment (Figures 3(i) and 3(j) and Table 3). Compared to patients diagnosed between 2000 and 2005, patients diagnosed with PCa between 2006 and 2016 had significantly reduced risks of other COD and PCSM (Figures 3(k) and 3(l) and Table 3).

## 4. Discussion

This study detailed the causes of death in patients with PCa for the years 2000 to 2016 and assessed the risk of death due to various causes compared to the general population. These data provide important guidance and help for the health maintenance of PCa patients. Our study found that the risk of death from all noncancer causes gradually decreased with each follow-up time period after diagnosis, although the results of the subgroup analysis are somewhat different. Specifically, noncancerous causes of death in patients with PCa account for 70% of total deaths, and the most common noncancerous causes of death are cardiovascular and cerebrovascular diseases and COPD. However, the risk of dying from noncancer causes within 5 years after PCa diagnosis was significantly higher than that of the general population. Conversely, patients with PCa have a similar or lower risk of dying from noncancerous causes of death within 5 to 10 years than the general population, except for other infectious diseases, Alzheimer's disease, and hyperextension without heart disease. Similarly, patients with PCa who have been around for more than 10 years have a similar or lower risk of dying from noncancer causes than the general population.

Although the overall survival of patients with PCa has improved over the past few decades, the proportion of noncancer causes of death has remained high [10, 12]. Studies have shown that cardiovascular disease and other cancers in men with early-stage prostate cancer and low-to-moderate-grade tumors are the main causes of death [10, 13]. In this study, the most common noncancer causes for patients with PCa were cardiovascular and cerebrovascular diseases and COPD, and the risk of death from cardiovascular and cerebrovascular diseases and COPD within five years after diagnosis of PCa was significantly higher than that of the general population. The reason may be related to PCa treatment, such as androgen deprivation therapy, and chemotherapy may be associated with an increased incidence of thromboembolic events, which may make men vulnerable to ischemic heart disease, stroke, and intracranial hemorrhage [14–16]. COPD causes increased levels of carbonyl stress and further DNA damage, induces proinflammatory signaling, and may increase the risk of PCa [17].

In addition, Epstein et al. described the time trend of specific causes of death in patients with PCa and found that the cumulative incidence of PCSM decreased during follow-up, while the cumulative incidences of death from ischemic heart disease and noncancer causes remained constant [7]. However, most of these findings were only analyzed in the PCa cohort, and compared with the general population, there is not much knowledge about the causes of noncancer deaths [6]. In this study, we found that the risk of death from noncancer causes within 5 years of diagnosis by PCa was significantly higher in the general population, in particular in other infectious diseases, septicemia, and suicide and self-inflicted injury. Infectious diseases and septicemia may occur due to neutropenia caused by systemic chemotherapy [18]. Bill-Axelson et al. found that the risk of committing suicide was twice as high among PCa with locally advanced or metastatic disease, compared with an age-matched male population [19]. And surgical treatment of PCa may cause erectile dysfunction and further lead to depression and suicide [20]. Therefore, the psychological burden brought by tumor diagnosis, treatment and monitoring, and the long recovery process bring uncontrolled pain to the patient and may lead to suicide [21]. Furthermore, patients over 65 years of age with PCa have a higher risk of dying from Alzheimer's disease than the general population, especially within 5 to 10 years after diagnosis of PCa. Studies have shown that Alzheimer's disease is a sporadic disease, but its incidence increases sharply with age [22, 23]. Some studies have shown that androgen deprivation therapy may cause cognitive dysfunction [24, 25], perhaps via impaired neuron growth and axonal regeneration or accumulation of abnormally folded *β*-amyloid protein [26]. However, patients diagnosed with PCa for more than 5 years have a lower risk of most noncancerous deaths than the general population. This implies that patients with PCa may have no effect on the long-term risk of death from noncancer causes.

Compared with the general population, distant-stage PCa has a higher risk of death from noncancer causes except for Alzheimer's disease, chronic liver disease, and aortic dissection. The deterioration of the advanced PCa itself and the patient's physical condition may affect the risk of death from noncancer causes. During the study period, compared with the general population, Black patients have a higher risk of death from noncancer causes except for COPD, suicide, chronic liver disease, accidents, and aortic dissection. Within 5 to 10 years after PCa diagnosis, Black patients appear to be at higher risk of heart disease, cerebrovascular disease, COPD, renal diseases, and septicemia compared with the general population. The risk of death of other races patients suffering from pneumonia and influenza and nephritis, nephrotic syndrome, and nephrosis is significantly higher within 1 to 5 years after cancer diagnosis. Explanations for these issues include changes in prostate-specific antigen screening recommendations, the stage at the time of diagnosis, and differences in socioeconomic status and geographic location field [27–29].

Moreover, we found that PCa patients with no treatment have a higher risk of death from suicide and heart and cerebrovascular diseases. This may be related to the patient's emotional state and financial burden [29, 30]. The risk of noncancer causes of death for patients undergoing RP±RT/CTx and RT/CTx is lower than that of the general population. Furthermore, compared with the general population, many noncancer causes of death have higher risk of death in patients with or without treatment within 1 to 5 years after diagnosis, whereas patients undergoing RP±RT/CTx are not at high risk of death from COPD, pneumonia and influenza, nephritis, nephrotic syndrome and nephrosis, septicemia, and atherosclerosis. Wallis et al. found that patients receiving RT had an increased risk of noncancer mortality and cardiovascular disease, which may be because of the fact that this study did not eliminate the impact of age factors compared with the general population [31]. And the average age of PCa patients receiving RT/CTx is higher, and the basic condition of the body is worse. Based on the above results, there are important findings. Attention should be paid not only to death due to PCa, but also, consideration is given to the risk of noncancer deaths that vary with the time since diagnosis, for timely and effective care and prevention.

There are some limitations to this study. First, the study was retrospective, and there was a lack of data regarding androgen deprivation therapy and chemotherapy, which could bias results. Secondly, some causes of death may not have been reported, and therefore, some may be listed under other causes in the registry. Finally, the SEER database does not capture several possible risk factors that affect the cause of death, such as socioeconomic status, geographic location, and germline mutation [32].

## 5. Conclusions

During each follow-up period following diagnosis, the majority of deaths occurred from noncancer causes in patients with PCa, with cardiovascular and cerebrovascular diseases and COPD being the most common causes. Moreover, the risk of death from all noncancerous causes in patients with PCa is gradually decreased, especially the risk of death from noncancer causes within 5 years after the diagnosis of PCa is significantly higher than that in the general population. In addition, the risk of dying from noncancer causes (COPD, suicide, chronic liver disease, and septicemia) is influenced by differences in treatment, ethnicity, and tumor stage. Therefore, these findings can provide important guidance for improving the survival and quality of life of patients with PCa.

## Figures and Tables

**Table 1:** Clinicopathological characteristics of patients with different death times and proportion of death after diagnosis of PCa.

Characteristic	Total	Timing of deaths after diagnosis									
		All deaths		<1 year		1 − 5 years		5 − 10 years		≥10 years	
		*N* (%)	Mean age at death (y)	*N* (%)	Mean age at death (y)	*N* (%)	Mean age at death (y)	*N* (%)	Mean age at death (y)	*N* (%)	Mean age at death (y)
Total	752352	180862		25004		70001		57150		28707	
Marital (%)											
Married	484508	104257 (100.0)	78.8	12111 (11.6)	77.0	37979 (36.4)	76.4	34742 (33.3)	79.8	19425 (18.6)	82.8
Widowed/divorced	89917	34089 (100.0)	79.7	6722 (19.7)	80.1	14061 (41.2)	78.1	9374 (27.5)	80.3	3932 (11.5)	82.9
Single	74066	18679 (100.0)	73.9	3385 (18.1)	72.2	7847 (42.0)	71.7	5162 (27.6)	75.9	2285 (12.2)	79.5
Unknown	103861	23837 (100.0)	80.2	2786 (11.7)	76.8	10114 (42.4)	78.4	7872 (33.0)	81.9	3065 (12.9)	84.6
Age (%)											
<50	23861	1778 (100.0)	50.9	223 (12.5)	46.0	800 (45.0)	47.9	494 (27.8)	53.8	261 (14.7)	58.9
50 − 64	302301	32270 (100.0)	64.8	3540 (11.0)	59.6	12780 (39.6)	61.8	10074 (31.2)	66.5	5876 (18.2)	71.7
65 − 74	273221	60457 (100.0)	76.1	5921 (9.8)	70.3	20980 (34.7)	72.7	20711 (34.3)	77.3	12845 (21.2)	82.3
≥75	152969	86357 (100.0)	86.1	15320 (17.7)	84.3	35441 (41.0)	84.7	25871 (30.0)	87.5	9725 (11.3)	90.7
Race, n (%)											
White	576407	141973 (100.0)	79.3	19162 (13.5)	78.1	54147 (38.1)	77.2	45430 (32.0)	80.4	23234 (16.4)	83.2
Black	113732	29146 (100.0)	74.7	4553 (15.6)	73.2	12039 (41.3)	72.7	8659 (29.7)	76.2	3895 (13.4)	79.5
Other	39111	8857 (100.0)	80.5	1139 (12.9)	78.4	3477 (39.3)	78.3	2781 (31.4)	81.7	1460 (16.5)	85.0
Unknown	23102	886 (100.0)	78.5	150 (16.9)	75.2	338 (38.1)	76.6	280 (31.6)	80.8	118 (13.3)	82.9
Year of diagnosis, n (%)											
2000–2005	247649	107914 (100.0)	79.7	10111 (9.4)	77.4	32834 (30.4)	77.3	36823 (34.1)	80.2	28146 (26.1)	82.8
2006–2016	504703	72948 (100.0)	77.0	14893 (20.4)	77.0	37167 (50.9)	75.8	20327 (27.9)	79.1	561 (.8)	81.3
Stage, n (%)											
Local	587431	123378 (100.0)	79.7	9314 (7.5)	75.9	43603 (35.3)	77.5	46260 (37.5)	80.5	24201 (19.6)	83.3
Regional	92553	15041 (100.0)	73.9	1138 (7.6)	75.1	5571 (37.0)	71.8	5373 (35.7)	73.6	2959 (19.7)	77.9
Distant	39192	28192 (100.0)	74.9	10570 (37.5)	76.5	14812 (52.5)	73.3	2408 (8.5)	76.7	402 (1.4)	80.5
Unknown	33176	14251 (100.0)	82.2	3982 (27.9)	82.5	6015 (42.2)	81.2	3109 (21.8)	82.9	1145 (8.0)	84.3
Grading, n (%)											
I	48184	3686 (100.0)	79.9	495 (13.4)	74.6	1212 (32.9)	77.5	1076 (29.2)	81.5	903 (24.5)	84.1
II	357337	71349 (100.0)	79.2	4588 (6.4)	73.7	21135 (29.6)	76.4	26981 (37.8)	79.8	18645 (26.1)	82.9
III	300115	79708 (100.0)	77.5	9725 (12.2)	75.3	36936 (46.3)	75.8	25323 (31.8)	79.3	7724 (9.7)	82.3
IV	1740	989 (100.0)	76.6	238 (24.1)	74.8	476 (48.1)	75.6	205 (20.7)	79.2	70 (7.1)	81.7
Unknown	44976	25130 (100.0)	80.5	9958 (39.6)	80.7	10242 (40.8)	79.2	3565 (14.2)	82.3	1365 (5.4)	83.4
Therapy, n (%)											
None	255913	98745 (100.0)	80.7	18667 (18.9)	78.9	43507 (44.1)	78.9	26786 (27.1)	82.9	9785 (9.9)	85.6
RP±RT/CT	244267	18563 (100.0)	71.5	920 (5.0)	64.4	4904 (26.4)	66.9	7293 (39.3)	71.3	5446 (29.3)	77.0
RT/CT	241813	59968 (100.0)	77.3	4444 (7.4)	71.6	20189 (33.7)	73.3	22213 (37.0)	78.7	13122 (21.9)	83.0
Unknown	10359	3586 (100.0)	81.1	973 (27.1)	81.1	1401 (39.1)	79.8	858 (23.9)	81.9	354 (9.9)	83.8

Mean age at death in years. RP: radical prostatectomy; RT: radiotherapy; W/D: widowed or divorced.

**Table 2:** Observed and SMRs by cause of death, stratified by years after diagnosis.

Cause of death	Total		<1 year		1 − 5 years		5 − 10 years		≥10 years	
	Observed	SMR (95%CI)	Observed	SMR (95%CI)	Observed	SMR (95%CI)	Observed	SMR (95%CI)	Observed	SMR (95%CI)
All causes of death	180862	0.95 (0.94 − 0.95)	25004	17.02 (16.79 − 17.25)	70001	3 (2.97 − 3.02)	57150	0.86 (0.85 − 0.86)	28707	0.3 (0.3 − 0.3)
Prostate cancer	53728	9.58 (9.5 − 9.66)	10507	124.06 (116.54 − 132.07)	26014	18.24 (17.93 − 18.55)	12670	7.31 (7.2 − 7.42)	4537	8.78 (8.67 − 8.9)
Noncancer causes of death	121510		13805		41975		42601		23129	
Diseases of heart	43096	0.81 (0.81 − 0.82)	4170	11.54 (11.2 − 11.9)	16016	2.4 (2.37 − 2.44)	15113	0.8 (0.79 − 0.81)	7797	0.29 (0.28 − 0.29)
Chronic obstructive pulmonary disease	8935	0.74 (0.73 − 0.76)	697	8.97 (8.33 − 9.66)	3319	2.24 (2.17 − 2.32)	3276	0.76 (0.74 − 0.79)	1643	0.26 (0.25 − 0.28)
Cerebrovascular diseases	8206	0.82 (0.81 − 0.84)	731	10.8 (10.05 − 11.61)	2926	2.35 (2.27 − 2.44)	2971	0.84 (0.81 − 0.87)	1578	0.31 (0.29 − 0.32)
Alzheimer′s	5130	1.1 (1.07 − 1.14)	212	6.2 (5.42 − 7.09)	1323	2.24 (2.12 − 2.36)	2079	1.27 (1.22 − 1.33)	1516	0.64 (0.6 − 0.67)
Diabetes mellitus	4605	0.8 (0.78 − 0.82)	400	10.77 (9.76 − 11.88)	1722	2.41 (2.3 − 2.53)	1663	0.81 (0.77 − 0.85)	820	0.28 (0.26 − 0.3)
Pneumonia and influenza	3637	0.79 (0.76 − 0.81)	303	9.23 (8.25 − 10.34)	1246	2.13 (2.02 − 2.26)	1371	0.84 (0.8 − 0.89)	717	0.3 (0.28 − 0.33)
Nephritis-NS and nephrosis	3046	0.78 (0.75 − 0.81)	227	8.55 (7.51 − 9.74)	1022	2.1 (1.97 − 2.23)	1153	0.83 (0.79 − 0.88)	644	0.32 (0.3 − 0.35)
Accidents and adverse effects	4258	0.86 (0.83 − 0.89)	391	11.47 (10.38 − 12.66)	1679	2.65 (2.52 − 2.78)	1454	0.82 (0.78 − 0.86)	734	0.29 (0.27 − 0.32)
Septicemia	2080	0.79 (0.75 − 0.82)	206	11.76 (10.26 − 13.48)	808	2.45 (2.29 − 2.63)	717	0.76 (0.71 − 0.82)	349	0.26 (0.23 − 0.29)
Hypertension without heart disease	1853	1.08 (1.03 − 1.13)	136	11.44 (9.67 − 13.53)	643	2.97 (2.75 − 3.21)	686	1.13 (1.04 − 1.21)	388	0.44 (0.4 − 0.49)
Other infectious diseases	1084	2.19 (2.06 − 2.33)	113	35.18 (29.25 − 42.3)	437	7.09 (6.46 − 7.79)	359	2.02 (1.83 − 2.24)	175	0.69(0.6 − 0.8)
In situ, benign, or unknown behavior neoplasms	500	0.39 (0.36 − 0.42)	42	4.94 (3.65 − 6.68)	167	1.05 (0.9 − 1.22)	194	0.42 (0.37 − 0.49)	97	0.15 (0.12 − 0.18)
Suicide and self − inflicted injury	1405	0.97 (0.92 − 1.02)	185	19.3 (16.71 − 22.3)	560	3 (2.76 − 3.26)	484	0.91 (0.84 − 1)	176	0.24 (0.21 − 0.28)
Chronic liver disease and cirrhosis	1116	0.6 (0.56 − 0.64)	89	7.36 (5.98 − 9.06)	520	2.17 (1.99 − 2.36)	331	0.48 (0.44 − 0.54)	176	0.19 (0.16 − 0.22)
Aortic aneurysm and dissection	788	0.66 (0.62 − 0.71)	95	12.37 (10.12 − 15.13)	332	2.26 (2.03 − 2.51)	258	0.61 (0.54 − 0.68)	103	0.17 (0.14 − 0.2)
Other diseases of arteries, arterioles, and capillaries	550	0.74 (0.68 − 0.81)	43	8.68 (6.44 − 11.71)	216	2.34 (2.05 − 2.68)	181	0.69 (0.59 − 0.8)	110	0.29 (0.24 − 0.35)
Atherosclerosis	549	0.82 (0.75 − 0.89)	51	10.61 (8.07 − 13.96)	196	2.31 (2 − 2.65)	198	0.84 (0.73 − 0.96)	104	0.3 (0.25 − 0.37)
Other cause of death	30672	—	5714	—	8843	—	10113	—	6002	—
Other cancers (non − prostate) causes of death	5624	0.13 (0.13 − 0.14)	692	2.59 (2.4 − 2.79)	2012	0.38 (0.37 − 0.4)	1879	0.12 (0.12 − 0.13)	1041	0.05 (0.05 − 0.05)

Bolded SMRs are significantly different from 1.00 (*P* < 0.05); the SMR, 95% CI, and *P* value were not calculated; —: the SMR representing the group is not calculated. NS: nephrotic syndrome.

**Table 3:** Other CODs and prostate-related hazard ratio of patients adjusted for demographic and clinical characteristics.

Variables	Other CODs		Prostate	
	sHR^a^	*P* value	sHR^a^	*P* value
Marital				
Married	1.00 (ref.)		1.00 (ref.)	
Widowed/divorced	1.40 (1.38 − 1.42)	<0.001	1.20 (1.17 − 1.23)	<0.001
Single	1.35 (1.32 − 1.38)	<0.001	1.12 (1.09 − 1.26)	<0.001
Unknown	1.17 (1.15 − 1.19)	<0.001	0.92 (0.89 − 0.95)	<0.001
Age				
< 50	1.00 (ref.)		1.00 (ref.)	
50 − 64	1.97 (1.83 − 2.12)	<0.001	1.03 (0.97 − 1.10)	0.357
65 − 74	4.66 (4.34 − 5.00)	<0.001	1.08 (1.01 − 1.15)	0.024
≥75	10.51 (9.78 − 11.29)	<0.001	1.46 (1.37 − 1.56)	<0.001
Race				
White	1.00 (ref.)		1.00 (ref.)	
Black	1.20 (1.18 − 1.22)	<0.001	1.06 (1.03 − 1.08)	<0.001
Other	0.86 (0.83 − 0.88)	<0.001	0.78 (0.75 − 0.81)	<0.001
Unknown	0.26 (0.24 − 0.28)	<0.001	0.13 (0.11 − 0.15)	<0.001
Year of diagnosis				
2000–2005	1.00 (ref.)		1.00 (ref.)	
2006–2016	0.71 (0.70 − 0.71)	<0.001	0.67 (0.66 − 0.69)	<0.001
Stage				
Local	1.00 (ref.)		1.00 (ref.)	
Regional	1.04 (1.01 − 1.06)	0.003	3.79 (3.67 − 3.90)	<0.001
Distant	0.58 (0.56 − 0.59)	<0.001	12.09 (11.80 − 12.39)	<0.001
Unknown	0.93 (0.90 − 0.96)	<0.001	3.05 (2.95 − 3.15)	<0.001
Grading				
I	1.00 (ref.)		1.00 (ref.)	
II	1.00 (0.97 − 1.04)	0.828	1.79 (1.60 − 2.00)	<0.001
III	1.12 (1.08 − 1.16)	<0.001	5.74 (5.13 − 6.42)	<0.001
IV	1.10 (0.99 − 1.23)	0.082	9.27 (7.94 − 10.82)	<0.001
Unknown	1.03 (0.99 − 1.07)	0.175	6.52 (5.82 − 7.31)	<0.001
Therapy				
None	1.00 (ref.)		1.00 (ref.)	
RP±RT/CT	0.36 (0.35 − 0.37)	<0.001	0.15 (0.14 − 0.15)	<0.001
RT/CT	0.69 (0.68 − 0.70)	<0.001	0.69 (0.67 − 0.70)	<0.001
Unknown	0.87 (0.83 − 0.91)	<0.001	1.20 (1.13 − 1.28)	<0.001

^a^Representing multivariable-adjusted competing risks, adjusted for marital, age at diagnosis, race, year of diagnosis, stage, grading, and therapy.

## Data Availability

Data are freely available upon request from the SEER database. Statistical code is available upon request to the corresponding author.

## Supplementary Materials

Supplementary Materials Supplementary Table 1: definition of each cause of death and corresponding codes in the ICD-10 of diseases and related health. Supplementary Table 2: standardized-mortality ratios (SMRs) for each cause of death following prostate cancer diagnosis in males aged younger than 50 years. Supplementary Table 3: standardized-mortality ratios (SMRs) for each cause of death following prostate cancer diagnosis in males aged 50-64 years. Supplementary Table 4: standardized-mortality ratios (SMRs) for each cause of death following prostate cancer diagnosis in males aged 65-75 years. Supplementary Table 5: standardized-mortality ratios (SMRs) for each cause of death following prostate cancer diagnosis in males older than 75 years. Supplementary Table 6: standardized-mortality ratios (SMRs) for each cause of death following prostate cancer diagnosis in married males. Supplementary Table 7: standardized-mortality ratios (SMRs) for each cause of death following prostate cancer diagnosis in widowed/divorced males. Supplementary Table 8: standardized-mortality ratios (SMRs) for each cause of death following prostate cancer diagnosis in single males. Supplementary Table 9: standardized-mortality ratios (SMRs) for each cause of death following prostate cancer diagnosis in White males. Supplementary Table 10: standardized-mortality ratios (SMRs) for each cause of death following prostate cancer diagnosis in Black males. Supplementary Table 11: standardized-mortality ratios (SMRs) for each cause of death following prostate cancer diagnosis in American Indian/Alaska Native Asian or Pacific Islander males. Supplementary Table 12: standardized-mortality ratios (SMRs) for each cause of death following local prostate cancer diagnosis. Supplementary Table 13: standardized-mortality ratios (SMRs) for each cause of death following regional prostate cancer diagnosis. Supplementary Table 14: standardized-mortality ratios (SMRs) for each cause of death following distant prostate cancer diagnosis. Supplementary Table 15: standardized-mortality ratios (SMRs) for each cause of death following prostate cancer diagnosis in males with grade I. Supplementary Table 16: standardized-mortality ratios (SMRs) for each cause of death following prostate cancer diagnosis in males with grade II. Supplementary Table 17: standardized-mortality ratios (SMRs) for each cause of death following prostate cancer diagnosis in males with grade III. Supplementary Table 18: standardized-mortality ratios (SMRs) for each cause of death following prostate cancer diagnosis in males with grade IV. Supplementary Table 19: standardized-mortality ratios (SMRs) for each cause of death following prostate cancer diagnosis in males with year of diagnosis between 2000 and 2005. Supplementary Table 20: standardized-mortality ratios (SMRs) for each cause of death following prostate cancer diagnosis in males with year of diagnosis between 2006 and 2016. Supplementary Table 21: Standardized-mortality ratios (SMRs) for each cause of death following prostate cancer diagnosis in males with nontreatment. Supplementary Table 22: standardized-mortality ratios (SMRs) for each cause of death following prostate cancer diagnosis in males with radical prostatectomy (RP)±radiotherapy/chemotherapy. Supplementary Table 23: standardized-mortality ratios (SMRs) for each cause of death following prostate cancer diagnosis in males with radiation±chemotherapy.
