# Supplementary material for: Causes of Death after Prostate Cancer Diagnosis: A Population-Based Study
Source: Oxid Med Cell Longev. 2022 Apr 23;2022:8145173. doi: 10.1155/2022/8145173 (PMC9056212; doi:10.1155/2022/8145173)
Supplement: Supplementary Materials — Supplementary Table 1: definition of each cause of death and corresponding codes in the ICD-10 of diseases and related health. Supplementary Table 2: standardized-mortality ratios (SMRs) for each cause of death following prostate cancer diagnosis in males aged younger than 50 years. Supplementary Table 3: standardized-mortality ratios (SMRs) for each cause of death following prostate cancer diagnosis in males aged 50-64 years. Supplementary Table 4: standardized-mortality ratios (SMRs) for each cause of death following prostate cancer diagnosis in males aged 65-75 years. Supplementary Table 5: standardized-mortality ratios (SMRs) for each cause of death following prostate cancer diagnosis in males older than 75 years. Supplementary Table 6: standardized-mortality ratios (SMRs) for each cause of death following prostate cancer diagnosis in married males. Supplementary Table 7: standardized-mortality ratios (SMRs) for each cause of death following prostate cancer diagnosis in widowed/divorced males. Supplementary Table 8: standardized-mortality ratios (SMRs) for each cause of death following prostate cancer diagnosis in single males. Supplementary Table 9: standardized-mortality ratios (SMRs) for each cause of death following prostate cancer diagnosis in White males. Supplementary Table 10: standardized-mortality ratios (SMRs) for each cause of death following prostate cancer diagnosis in Black males. Supplementary Table 11: standardized-mortality ratios (SMRs) for each cause of death following prostate cancer diagnosis in American Indian/Alaska Native Asian or Pacific Islander males. Supplementary Table 12: standardized-mortality ratios (SMRs) for each cause of death following local prostate cancer diagnosis. Supplementary Table 13: standardized-mortality ratios (SMRs) for each cause of death following regional prostate cancer diagnosis. Supplementary Table 14: standardized-mortality ratios (SMRs) for each cause of death following distant prostate cancer diagnosis. S [file 8145173.f1.docx]

**Supplementary Table 1.** Definition of each cause of death and corresponding codes in the ICD-10 of Diseases and Related Health.

| Cause of death | ICD-10 corresponding codes | Cause of death definition |
| --- | --- | --- |
| All causes of death |  |  |
| Non-cancer causes of death |  |  |
| Diseases of Heart | I00-I02 | Acute rheumatic fever |
|  | I05-I09 | Chronic rheumatic heart diseases |
|  | I11 | Hypertensive heart disease |
|  | I13 | Hypertensive heart and renal disease |
|  | I20-I25 | Ischemic heart diseases |
|  | I26-I28 | Pulmonary heart disease and diseases of pulmonary circulation |
|  | I30-I32 | Diseases of pericardium |
|  | I33 | Acute and subacute endocarditis |
|  | I34-I39 | Nonrheumatic valve disorders |
|  | I40-I41 | Myocarditis |
|  | I42-I43 | Cardiomyopathy |
|  | I44-I45 | Conduction disorders |
|  | I46 | Cardiac arrest |
|  | I47-I49 | Arrythmias |
|  | I50 | Heart failure |
|  | I51 | Complications and ill-defined descriptions of heart disease |
| Chronic Obstructive Pulmonary Disease | J40-J42 | Bronchitis |
|  | J43 | Emphysema |
|  | J44 | Other chronic obstructive pulmonary disease |
|  | J45-J46 | Asthma or Status asthmaticus |
|  | J47 | Bronchiectasis |
| Cerebrovascular Diseases | I60-I62 | Nontraumatic intracranial haemorrhage |
|  | I63 | Cerebral infarction |
|  | I64 | Stroke, not specified as haemorrhage or infarction |
|  | I65-I66 | Occlusion and stenosis of precerebral/cerebral arteries, not resulting in cerebral infarction |
|  | I67-I69 | Other cerebrovascular diseases or Sequelae of cerebrovascular disease |
| Alzheimer’s | G30 | Alzheimer disease |
| Diabetes Mellitus | E10-E14 | Diabetes mellitus |
| Pneumonia and Influenza | J09-J18 | Influenza and pneumonia |
| Nephritis, Nephrotic Syndrome and Nephrosis | N00-N07 | Glomerular diseases |
|  | N17-N19 | Renal failure |
|  | N25 | Disorders resulting from impaired renal tubular function |
|  | N26 | Unspecified contracted kidney |
|  | N27 | Small kidney of unknown cause |
| Accidents and Adverse Effects | V01-V99 | Transport accidents |
|  | W00-X59 | Other external causes of accidental injury |
|  | Y85-Y86 | Sequelae of transport accidents or other accidents |
| Septicemia | A40-A41 | Sepsis |
| Hypertension without Heart Disease | I10 | Essential (primary) hypertension |
|  | I12 | Hypertensive renal disease |
| Other Infectious Diseases | B99 |  |
| In situ, benign or unknown behavior neoplasms | D00-D09 | In situ neoplasms |
| Suicide and Self-Inflicted Injury | X60-X84 | Intentional self-harm |
| Chronic Liver Disease and Cirrhosis | K70 | Alcoholic liver disease |
|  | K73 | Chronic hepatitis |
|  | K74 | Fibrosis and cirrhosis of liver |
| Aortic Aneurysm and Dissection | I71 | Aortic Aneurysm and Dissection |
| Other Diseases of Arteries, Arterioles, Capillaries | I72-I73 | Other aneurysm and dissection or other peripheral vascular diseases |
| Atherosclerosis | I70 | Atherosclerosis |
| Other cancers (Non-prostate) causes of death |  |  |

**Supplementary Table 2.** Standardized-mortality ratios (SMRs) for each cause of death following prostate cancer diagnosis in males aged younger than 50 years.

| Cause of death | **Total** | | **< 1 Year** | | **1-5 Years** | | **5-10 Years** | | **≥10 Years** | |
| --- | --- | --- | --- | --- | --- | --- | --- | --- | --- | --- |
|  | Observed | SMR | Observed | SMR | Observed | SMR | Observed | SMR | Observed | SMR |
|  |  | (95% CI) |  | (95% CI) |  | (95% CI) |  | (95% CI) |  | (95% CI) |
| All causes of death | 1759 | **1.48(1.41-1.55)** | 204 | **79.47(69.28-91.16)** | 800 | **10.6(9.89-11.36)** | 494 | **1.43(1.31-1.56)** | 261 | **0.34(0.3-0.39)** |
| Non-cancer causes of death |  |  |  |  |  |  |  |  |  |  |
| Diseases of Heart | 225 | **0.77(0.68-0.88)** | 18 | **32.37(20.39-51.37)** | 74 | **4.3(3.42-5.4)** | 83 | 1.01(0.81-1.25) | 50 | **0.26(0.2-0.35)** |
| Chronic Obstructive Pulmonary Disease | 20 | **0.68(0.44-1.05)** | 1 | — | 3 | — | 8 | — | 8 | — |
| Cerebrovascular Diseases | 31 | 0.85(0.6-1.21) | 4 | — | 8 | — | 8 | — | 11 | **0.46(0.25-0.83)** |
| Alzheimer’s |  |  |  | — |  | — |  | — |  |  |
| Diabetes Mellitus | 24 | **0.59(0.4-0.89)** | 1 | — | 5 | — | 9 | — | 9 | — |
| Pneumonia and Influenza | 11 | **0.82(0.45-1.48)** | 1 | — | 1 | — | 3 | — | 6 | — |
| Nephritis- Nephrotic Syndrome and Nephrosis | 14 | **0.99(0.59-1.67)** | 1 | — | 3 | — | 6 | — | 4 | — |
| Accidents and Adverse Effects | 58 | **0.51(0.4-0.66)** | 8 | — | 19 | **1.89(1.21-2.97)** | 20 | **0.53(0.34-0.82)** | 11 | **0.17(0.09-0.31)** |
| Septicemia |  |  | 2 | — | 8 | — | 6 | — |  |  |
| Hypertension without Heart Disease | 14 | 1.56(0.92-2.63) |  |  | 6 | — | 5 | — | 3 | — |
| Other Infectious Diseases | 21 | **4.35(2.84-6.67)** | 2 | — | 10 | **30.57(16.45-56.82)** | 8 | — | 1 | — |
| In situ- benign or unknown behavior neoplasms | 3 | — |  |  | 2 | — |  |  | 1 | — |
| Suicide and Self-Inflicted Injury | 43 | 0.84(0.62-1.13) | 6 | — | 17 | **3.81(2.37-6.14)** | 13 | 0.76(0.44-1.31) | 7 | — |
| Chronic Liver Disease and Cirrhosis | 36 | **0.66(0.48-0.91)** | 1 | — | 17 | **4.48(2.79-7.21)** | 8 | — | 10 | **0.3(0.16-0.55)** |
| Aortic Aneurysm and Dissection | 3 | — |  |  |  |  | 2 | — | 1 | — |
| Other Diseases of Arteries- Arterioles- Capillaries | 1 | — |  |  |  |  | 1 | — |  |  |
| Atherosclerosis | 4 | — |  |  | 2 | — | 2 | — |  |  |
| Other cancers (Non-prostate) causes of death | 84 | **0.29(0.23-0.35)** | 14 | **30.92(18.31-52.21)** | 37 | **2.44(1.77-3.37)** | 16 | **0.2(0.12-0.33)** | 17 | **0.09(0.05-0.14)** |

Bolded SMRs are significantly different from 1.00 (P < .05). A blank indicates that because of 0 observed deaths, the SMR, 95% CI, and P value were not calculated.

**Supplementary Table 3.** Standardized-mortality ratios (SMRs) for each cause of death following prostate cancer diagnosis in males aged 50-64 years.

| Cause of death | **Total** | | **< 1 Year** | | **1-5 Years** | | **5-10 Years** | | **≥10 Years** | |
| --- | --- | --- | --- | --- | --- | --- | --- | --- | --- | --- |
|  | Observed | SMR | Observed | SMR | Observed | SMR | Observed | SMR | Observed | SMR |
|  |  | (95% CI) |  | (95% CI) |  | (95% CI) |  | (95% CI) |  | (95% CI) |
| All causes of death | 31948 | **0.9(0.89-0.91)** | 3218 | **26.76(25.85-27.7)** | 12780 | **4.5(4.42-4.58)** | 10074 | **0.93(0.91-0.95)** | 5876 | **0.27(0.26-0.28)** |
| Non-cancer causes of death |  |  |  |  |  |  |  |  |  |  |
| Diseases of Heart | 6447 | **0.69(0.68-0.71)** | 540 | **17.25(15.85-18.77)** | 2274 | **3.08(2.95-3.21)** | 2245 | **0.79(0.76-0.83)** | 1388 | **0.24(0.23-0.26)** |
| Chronic Obstructive Pulmonary Disease | 1214 | **0.61(0.58-0.65)** | 54 | **11.37(8.71-14.84)** | 331 | **2.63(2.37-2.93)** | 477 | **0.86(0.79-0.94)** | 352 | **0.27(0.24-0.3)** |
| Cerebrovascular Diseases | 957 | **0.69(0.65-0.74)** | 83 | **20.54(16.57-25.47)** | 303 | **3.07(2.75-3.44)** | 334 | **0.84(0.75-0.94)** | 237 | **0.27(0.24-0.3)** |
| Alzheimer’s | 3 | — | 2 | — | 1 | — | 0 | — | 2 | — |
| Diabetes Mellitus | 906 | **0.67(0.63-0.72)** | 77 | **16.84(13.47-21.05)** | 298 | **2.74(2.45-3.07)** | 331 | **0.79(0.71-0.88)** | 200 | **0.24(0.21-0.28)** |
| Pneumonia and Influenza | 302 | **0.6(0.53-0.67)** | 15 | **10.33(6.23-17.14)** | 90 | **2.54(2.06-3.12)** | 112 | **0.78(0.65-0.94)** | 85 | **0.26(0.21-0.32)** |
| Nephritis- Nephrotic Syndrome and Nephrosis | 418 | **0.73(0.67-0.81)** | 23 | **13.87(9.22-20.87)** | 119 | **2.92(2.44-3.49)** | 152 | 0.92(0.78-1.08) | 124 | **0.34(0.29-0.41)** |
| Accidents and Adverse Effects | 985 | **0.78(0.73-0.83)** | 87 | **13.9(11.27-17.15)** | 409 | **3.1(2.82-3.42)** | 318 | **0.75(0.67-0.83)** | 171 | **0.24(0.21-0.28)** |
| Septicemia | 16 | 1.14(0.7-1.86) | 24 | **15.37(10.3-22.93)** | 110 | **2.93(2.43-3.53)** | 134 | 0.91(0.77-1.08) | 80 | **0.26(0.21-0.33)** |
| Hypertension without Heart Disease | 307 | 1.07(0.96-1.2) | 21 | **21.43(13.97-32.87)** | 124 | **5.38(4.52-6.42)** | 89 | 1.02(0.83-1.25) | 73 | **0.42(0.33-0.53)** |
| Other Infectious Diseases | 268 | **2.27(2.01-2.56)** | 32 | **74.69(52.82-105)** | 106 | **10.68(8.83-12.92)** | 84 | **2.28(1.84-2.82)** | 46 | **0.65(0.49-0.87)** |
| In situ- benign or unknown behavior neoplasms | 64 | **0.33(0.26-0.42)** | 3 | — | 15 | 1.13(0.68-1.87) | 28 | **0.51(0.35-0.73)** | 18 | **0.14(0.09-0.23)** |
| Suicide and Self-Inflicted Injury | 481 | **0.88(0.8-0.96)** | 54 | **18.9(14.48-24.68)** | 198 | **3.33(2.89-3.82)** | 171 | 0.91(0.78-1.05) | 58 | **0.19(0.15-0.25)** |
| Chronic Liver Disease and Cirrhosis | 434 | **0.51(0.47-0.56)** | 29 | **6.83(4.75-9.83)** | 188 | **2.08(1.81-2.41)** | 141 | **0.48(0.41-0.57)** | 76 | **0.17(0.13-0.21)** |
| Aortic Aneurysm and Dissection | 133 | **0.58(0.49-0.69)** | 13 | **19.75(11.47-34.01)** | 50 | **3.06(2.32-4.04)** | 44 | **0.66(0.49-0.89)** | 26 | **0.18(0.12-0.26)** |
| Other Diseases of Arteries- Arterioles- Capillaries | 86 | **0.71(0.58-0.88)** | 7 | — | 27 | **3.2(2.19-4.66)** | 30 | 0.85(0.6-1.22) | 22 | **0.29(0.19-0.43)** |
| Atherosclerosis | 33 | **0.5(0.35-0.7)** | 2 | — | 8 | — | 13 | 0.71(0.41-1.22) | 10 | **0.23(0.12-0.43)** |
| Other cancers (Non-prostate) causes of death | 1246 | **0.11(0.11-0.12)** | 104 | **2.86(2.36-3.47)** | 387 | **0.44(0.4-0.49)** | 436 | **0.13(0.12-0.14)** | 319 | **0.05(0.04-0.05)** |

Bolded SMRs are significantly different from 1.00 (P < .05). A blank indicates that because of 0 observed deaths, the SMR, 95% CI, and P value were not calculated.

**Supplementary Table 4.**  Standardized-mortality ratios (SMRs) for each cause of death following prostate cancer diagnosis in males aged 65-75 years.

| Cause of death | **Total** | | **< 1 Year** | | **1-5 Years** | | **5-10 Years** | | **≥10 Years** | |
| --- | --- | --- | --- | --- | --- | --- | --- | --- | --- | --- |
|  | Observed | SMR | Observed | SMR | Observed | SMR | Observed | SMR | Observed | SMR |
|  |  | (95% CI) |  | (95% CI) |  | (95% CI) |  | (95% CI) |  | (95% CI) |
| All causes of death | 59797 | **0.83(0.82-0.84)** | 5261 | **18.51(18.02-19.02)** | 20980 | **3.2(3.16-3.25)** | 20711 | **0.91(0.9-0.93)** | 12845 | **0.3(0.3-0.31)** |
| Non-cancer causes of death |  |  |  |  |  |  |  |  |  |  |
| Diseases of Heart | 14671 | **0.75(0.74-0.76)** | 1157 | **15.69(14.81-16.62)** | 4857 | **2.84(2.76-2.92)** | 5260 | **0.88(0.85-0.9)** | 3397 | **0.29(0.28-0.3)** |
| Chronic Obstructive Pulmonary Disease | 3584 | **0.7(0.68-0.72)** | 193 | **10.03(8.71-11.55)** | 1207 | **2.64(2.49-2.79)** | 1396 | **0.85(0.81-0.9)** | 788 | **0.26(0.24-0.28)** |
| Cerebrovascular Diseases | 2897 | **0.78(0.75-0.81)** | 168 | **13.94(11.98-16.21)** | 868 | **2.96(2.77-3.16)** | 1118 | 1.01(0.95-1.07) | 743 | **0.32(0.3-0.35)** |
| Alzheimer’s | 212 | 1.09(0.95-1.25) | 16 | **7.29(4.47-11.9)** | 29 | **4.2(2.92-6.05)** | 81 | **2.06(1.66-2.56)** | 100 | **0.67(0.55-0.82)** |
| Diabetes Mellitus | 1928 | **0.81(0.77-0.85)** | 133 | **12.44(10.49-14.74)** | 656 | **2.74(2.54-2.96)** | 708 | **0.9(0.83-0.96)** | 431 | **0.32(0.29-0.35)** |
| Pneumonia and Influenza | 1104 | **0.72(0.68-0.76)** | 46 | **10.37(7.77-13.85)** | 308 | **2.8(2.5-3.13)** | 457 | 1.06(0.97-1.17) | 293 | **0.3(0.26-0.33)** |
| Nephritis- Nephrotic Syndrome and Nephrosis | 1124 | **0.78(0.73-0.82)** | 66 | **13.26(10.42-16.88)** | 316 | **2.66(2.38-2.96)** | 432 | 0.99(0.9-1.09) | 310 | **0.35(0.31-0.39)** |
| Accidents and Adverse Effects | 1440 | **0.86(0.82-0.91)** | 115 | **16.13(13.44-19.36)** | 509 | **3.21(2.94-3.5)** | 496 | 0.94(0.86-1.03) | 320 | **0.33(0.29-0.36)** |
| Septicemia | 348 | **0.71(0.64-0.79)** | 58 | **14.28(11.04-18.47)** | 259 | **2.75(2.43-3.1)** | 271 | **0.82(0.73-0.92)** | 164 | **0.27(0.23-0.31)** |
| Hypertension without Heart Disease | 628 | 1.05(0.97-1.13) | 42 | **18.88(13.95-25.54)** | 180 | **3.5(3.03-4.05)** | 238 | **1.32(1.16-1.5)** | 168 | **0.46(0.39-0.53)** |
| Other Infectious Diseases | 381 | **1.9(1.71-2.1)** | 30 | **33.45(23.39-47.84)** | 138 | **6.86(5.81-8.11)** | 136 | **2.05(1.73-2.42)** | 77 | **0.68(0.54-0.85)** |
| In situ- benign or unknown behavior neoplasms | 169 | **0.33(0.29-0.39)** | 11 | **6.26(3.47-11.31)** | 55 | **1.3(1-1.69)** | 61 | **0.39(0.3-0.5)** | 42 | **0.14(0.1-0.18)** |
| Suicide and Self-Inflicted Injury | 532 | 1.01(0.93-1.1) | 74 | **25.81(20.55-32.42)** | 193 | **3.18(2.76-3.66)** | 177 | 0.97(0.83-1.12) | 88 | **0.31(0.26-0.39)** |
| Chronic Liver Disease and Cirrhosis | 445 | **0.65(0.59-0.71)** | 40 | **8.99(6.59-12.25)** | 208 | **2.3(2.01-2.63)** | 126 | **0.5(0.42-0.6)** | 71 | **0.21(0.17-0.26)** |
| Aortic Aneurysm and Dissection | 305 | **0.61(0.54-0.68)** | 22 | **10.93(7.2-16.6)** | 123 | **2.62(2.2-3.13)** | 110 | **0.67(0.56-0.81)** | 50 | **0.17(0.13-0.23)** |
| Other Diseases of Arteries- Arterioles- Capillaries | 193 | **0.67(0.58-0.77)** | 9 | — | 68 | **2.68(2.11-3.4)** | 58 | **0.64(0.5-0.83)** | 58 | **0.34(0.26-0.44)** |
| Atherosclerosis | 180 | **0.82(0.71-0.95)** | 11 | **18.02(9.98-32.53)** | 49 | **3.19(2.41-4.22)** | 73 | 1.2(0.95-1.5) | 47 | **0.33(0.25-0.44)** |
| Other cancers (Non-prostate) causes of death | 2090 | **0.11(0.11-0.12)** | 181 | **2.04(1.76-2.36)** | 640 | **0.33(0.3-0.35)** | 801 | **0.13(0.12-0.14)** | 468 | **0.05(0.04-0.05)** |

Bolded SMRs are significantly different from 1.00 (P < .05). A blank indicates that because of 0 observed deaths, the SMR, 95% CI, and P value were not calculated.

**Supplementary Table 5.** Standardized-mortality ratios (SMRs) for each cause of death following prostate cancer diagnosis in males older than 75 years.

| Cause of death | **Total** | | **< 1 Year** | | **1-5 Years** | | **5-10 Years** | | **≥10 Years** | |
| --- | --- | --- | --- | --- | --- | --- | --- | --- | --- | --- |
|  | Observed | SMR | Observed | SMR | Observed | SMR | Observed | SMR | Observed | SMR |
|  |  | (95% CI) |  | (95% CI) |  | (95% CI) |  | (95% CI) |  | (95% CI) |
| All causes of death | 83618 | **1.07(1.06-1.08)** | 12581 | **14.94(14.68-15.2)** | 35441 | **2.55(2.52-2.58)** | 25871 | **0.79(0.78-0.8)** | 9725 | **0.32(0.31-0.32)** |
| Non-cancer causes of death |  |  |  |  |  |  |  |  |  |  |
| Diseases of Heart | 21753 | **0.91(0.9-0.92)** | 2455 | **9.6(9.23-9.99)** | 8811 | **2.1(2.06-2.14)** | 7525 | **0.75(0.74-0.77)** | 2962 | **0.31(0.3-0.32)** |
| Chronic Obstructive Pulmonary Disease | 4117 | **0.84(0.81-0.86)** | 449 | **8.37(7.63-9.18)** | 1778 | **1.99(1.9-2.08)** | 1395 | **0.67(0.63-0.7)** | 495 | **0.26(0.24-0.29)** |
| Cerebrovascular Diseases | 4321 | **0.89(0.87-0.92)** | 476 | **9.24(8.45-10.11)** | 1747 | **2.06(1.96-2.16)** | 1511 | **0.75(0.71-0.79)** | 587 | **0.31(0.28-0.33)** |
| Alzheimer’s | 1600 | **1.15(1.1-1.21)** | 194 | **6.1(5.3-7.02)** | 238 | **3.54(3.12-4.03)** | 641 | **1.92(1.77-2.07)** | 705 | **0.72(0.66-0.77)** |
| Diabetes Mellitus | 1747 | **0.88(0.84-0.92)** | 189 | **8.67(7.52-10)** | 763 | **2.1(1.95-2.25)** | 615 | **0.73(0.67-0.79)** | 180 | **0.24(0.21-0.28)** |
| Pneumonia and Influenza | 2220 | **0.87(0.83-0.91)** | 241 | **8.96(7.9-10.17)** | 847 | **1.94(1.81-2.07)** | 799 | **0.76(0.71-0.81)** | 333 | **0.32(0.29-0.36)** |
| Nephritis- Nephrotic Syndrome and Nephrosis | 1490 | **0.8(0.76-0.84)** | 137 | **6.89(5.83-8.14)** | 584 | **1.79(1.65-1.94)** | 563 | **0.72(0.67-0.78)** | 206 | **0.28(0.24-0.32)** |
| Accidents and Adverse Effects | 1775 | **0.94(0.89-0.98)** | 181 | **8.91(7.7-10.31)** | 742 | **2.22(2.07-2.39)** | 620 | **0.78(0.72-0.85)** | 232 | **0.31(0.27-0.35)** |
| Septicemia | 752 | **0.72(0.67-0.77)** | 122 | **10.28(8.61-12.28)** | 431 | **2.19(1.99-2.41)** | 306 | **0.66(0.59-0.74)** | 105 | **0.25(0.2-0.3)** |
| Hypertension without Heart Disease | 904 | **1.11(1.04-1.18)** | 73 | **8.42(6.69-10.59)** | 333 | **2.35(2.11-2.62)** | 354 | 1.05(0.94-1.16) | 144 | **0.44(0.37-0.52)** |
| Other Infectious Diseases | 414 | **2.42(2.2-2.67)** | 49 | **26.12(19.74-34.56)** | 183 | **5.85(5.07-6.77)** | 131 | **1.8(1.52-2.14)** | 51 | **0.78(0.6-1.03)** |
| In situ- benign or unknown behavior neoplasms | 264 | **0.46(0.4-0.52)** | 28 | **4.51(3.11-6.53)** | 95 | **0.92(0.75-1.13)** | 105 | **0.43(0.36-0.52)** | 36 | **0.16(0.12-0.22)** |
| Suicide and Self-Inflicted Injury | 349 | 1.06(0.96-1.18) | 51 | **13.84(10.52-18.21)** | 152 | **2.45(2.09-2.88)** | 123 | 0.87(0.73-1.04) | 23 | **0.19(0.13-0.29)** |
| Chronic Liver Disease and Cirrhosis | 201 | **0.73(0.63-0.83)** | 19 | **5.81(3.71-9.11)** | 107 | **1.93(1.6-2.34)** | 56 | **0.46(0.36-0.6)** | 19 | **0.19(0.12-0.31)** |
| Aortic Aneurysm and Dissection | 347 | **0.76(0.68-0.84)** | 60 | **12.01(9.32-15.47)** | 159 | **1.91(1.63-2.23)** | 102 | **0.52(0.43-0.64)** | 26 | **0.15(0.1-0.22)** |
| Other Diseases of Arteries- Arterioles- Capillaries | 270 | **0.82(0.73-0.93)** | 27 | **7.64(5.24-11.13)** | 121 | **2.08(1.74-2.48)** | 92 | **0.67(0.55-0.82)** | 30 | **0.23(0.16-0.33)** |
| Atherosclerosis | 332 | **0.87(0.78-0.97)** | 38 | **9.45(6.87-12.98)** | 137 | **2.1(1.78-2.48)** | 110 | **0.7(0.58-0.84)** | 47 | **0.3(0.23-0.4)** |
| Other cancers (Non-prostate) causes of death | 2204 | **0.17(0.17-0.18)** | 393 | **2.77(2.51-3.06)** | 948 | **0.4(0.37-0.42)** | 626 | **0.11(0.11-0.12)** | 237 | **0.05(0.04-0.06)** |

Bolded SMRs are significantly different from 1.00 (P < .05). A blank indicates that because of 0 observed deaths, the SMR, 95% CI, and P value were not calculated.

**Supplementary Table 6.**  Standardized-mortality ratios (SMRs) for each cause of death following prostate cancer diagnosis in married males.

| Cause of death | **Total** | | **< 1 Year** | | **1-5 Years** | | **5-10 Years** | | **≥10 Years** | |
| --- | --- | --- | --- | --- | --- | --- | --- | --- | --- | --- |
|  | Observed | SMR | Observed | SMR | Observed | SMR | Observed | SMR | Observed | SMR |
|  |  | (95% CI) |  | (95% CI) |  | (95% CI) |  | (95% CI) |  | (95% CI) |
| All causes of death | 102554 | **0.81(0.81-0.82)** | 10408 | **15.55(15.25-15.85)** | 37979 | **2.86(2.83-2.88)** | 34742 | **0.82(0.81-0.83)** | 19425 | **0.28(0.28-0.28)** |
| Non-cancer causes of death |  |  |  |  |  |  |  |  |  |  |
| Diseases of Heart | 24811 | **0.7(0.69-0.71)** | 1987 | **10.38(9.93-10.84)** | 8498 | **2.26(2.21-2.31)** | 9067 | **0.76(0.74-0.77)** | 5259 | **0.27(0.26-0.28)** |
| Chronic Obstructive Pulmonary Disease | 4993 | **0.61(0.59-0.63)** | 318 | **7.57(6.79-8.45)** | 1690 | **1.99(1.9-2.09)** | 1920 | **0.7(0.67-0.73)** | 1065 | **0.24(0.22-0.25)** |
| Cerebrovascular Diseases | 4909 | **0.74(0.72-0.76)** | 381 | **10.7(9.68-11.83)** | 1615 | **2.32(2.21-2.43)** | 1821 | **0.82(0.78-0.86)** | 1092 | **0.3(0.28-0.31)** |
| Alzheimer’s | 3286 | **1.09(1.05-1.12)** | 108 | **6.31(5.23-7.62)** | 767 | **2.43(2.26-2.6)** | 1333 | **1.34(1.27-1.42)** | 1078 | **0.63(0.6-0.67)** |
| Diabetes Mellitus | 2679 | **0.68(0.66-0.71)** | 213 | **10.45(9.13-11.95)** | 969 | **2.34(2.19-2.49)** | 955 | **0.72(0.67-0.76)** | 542 | **0.25(0.23-0.27)** |
| Pneumonia and Influenza | 2075 | **0.68(0.65-0.71)** | 156 | **9.25(7.91-10.82)** | 626 | **1.95(1.81-2.11)** | 815 | **0.81(0.75-0.86)** | 478 | **0.28(0.26-0.31)** |
| Nephritis- Nephrotic Syndrome and Nephrosis | 1730 | **0.66(0.63-0.7)** | 107 | **7.65(6.33-9.25)** | 513 | **1.88(1.72-2.04)** | 682 | **0.78(0.72-0.84)** | 428 | **0.3(0.27-0.33)** |
| Accidents and Adverse Effects | 2478 | **0.74(0.71-0.77)** | 168 | **9.16(7.88-10.66)** | 926 | **2.55(2.39-2.72)** | 897 | **0.79(0.74-0.84)** | 487 | **0.27(0.25-0.29)** |
| Septicemia | 1163 | **0.65(0.61-0.69)** | 105 | **11.15(9.21-13.51)** | 406 | **2.16(1.96-2.38)** | 428 | **0.71(0.65-0.78)** | 224 | **0.23(0.2-0.26)** |
| Hypertension without Heart Disease | 1022 | **0.89(0.84-0.95)** | 55 | **8.81(6.76-11.47)** | 320 | **2.64(2.37-2.95)** | 394 | 1.03(0.93-1.13) | 253 | **0.4(0.35-0.45)** |
| Other Infectious Diseases | 553 | **1.64(1.51-1.79)** | 54 | **30.68(23.5-40.06)** | 209 | **5.85(5.11-6.7)** | 183 | **1.6(1.38-1.85)** | 107 | **0.58(0.48-0.7)** |
| In situ- benign or unknown behavior neoplasms | 312 | **0.36(0.32-0.4)** | 18 | **3.97(2.5-6.3)** | 102 | 1.13(0.93-1.38) | 122 | **0.42(0.35-0.5)** | 70 | **0.15(0.12-0.18)** |
| Suicide and Self-Inflicted Injury | 754 | **0.76(0.7-0.81)** | 89 | **16.52(13.42-20.34)** | 276 | **2.49(2.22-2.81)** | 273 | **0.79(0.7-0.89)** | 116 | **0.22(0.18-0.26)** |
| Chronic Liver Disease and Cirrhosis | 628 | **0.49(0.45-0.53)** | 44 | **6.34(4.72-8.52)** | 268 | **1.86(1.65-2.09)** | 200 | **0.44(0.38-0.51)** | 116 | **0.17(0.14-0.2)** |
| Aortic Aneurysm and Dissection | 496 | **0.61(0.56-0.67)** | 41 | **9.81(7.22-13.33)** | 199 | **2.35(2.04-2.7)** | 178 | **0.65(0.56-0.75)** | 78 | **0.17(0.14-0.22)** |
| Other Diseases of Arteries- Arterioles- Capillaries | 315 | **0.63(0.57-0.71)** | 23 | **8.72(5.8-13.13)** | 112 | **2.15(1.78-2.58)** | 107 | **0.64(0.53-0.77)** | 73 | **0.27(0.21-0.33)** |
| Atherosclerosis | 287 | **0.65(0.58-0.73)** | 26 | **10.58(7.2-15.54)** | 89 | **1.92(1.56-2.36)** | 111 | **0.76(0.63-0.91)** | 61 | **0.25(0.19-0.32)** |
| Other cancers (Non-prostate) causes of death | 3342 | **0.11(0.11-0.12)** | 353 | **2.37(2.13-2.63)** | 1107 | **0.36(0.34-0.38)** | 1165 | **0.12(0.11-0.12)** | 717 | **0.04(0.04-0.05)** |

Bolded SMRs are significantly different from 1.00 (P < .05). A blank indicates that because of 0 observed deaths, the SMR, 95% CI, and P value were not calculated.

**Supplementary Table 7.**  Standardized-mortality ratios (SMRs) for each cause of death following prostate cancer diagnosis in Widowed/Divorced males.

| Cause of death | **Total** | | **< 1 Year** | | **1-5 Years** | | **5-10 Years** | | **≥10 Years** | |
| --- | --- | --- | --- | --- | --- | --- | --- | --- | --- | --- |
|  | Observed | SMR | Observed | SMR | Observed | SMR | Observed | SMR | Observed | SMR |
|  |  | (95% CI) |  | (95% CI) |  | (95% CI) |  | (95% CI) |  | (95% CI) |
| All causes of death | 32905 | **1.4(1.38-1.41)** | 5538 | **19.76(19.25-20.29)** | 14061 | **3.54(3.48-3.6)** | 9374 | **1.05(1.03-1.08)** | 3932 | **0.38(0.37-0.39)** |
| Non-cancer causes of death |  |  |  |  |  |  |  |  |  |  |
| Diseases of Heart | 7939 | **1.17(1.14-1.19)** | 1059 | **12.55(11.82-13.33)** | 3263 | **2.79(2.7-2.89)** | 2525 | **0.98(0.94-1.02)** | 1092 | **0.37(0.35-0.39)** |
| Chronic Obstructive Pulmonary Disease | 1793 | **1.19(1.14-1.25)** | 194 | **11.32(9.83-13.03)** | 736 | **2.96(2.76-3.18)** | 616 | **1.08(1-1.17)** | 247 | **0.37(0.33-0.42)** |
| Cerebrovascular Diseases | 1369 | **1.06(1-1.11)** | 149 | **9.13(7.77-10.72)** | 534 | **2.38(2.19-2.6)** | 480 | 0.98(0.9-1.07) | 206 | **0.37(0.33-0.43)** |
| Alzheimer’s | 766 | **1.16(1.08-1.24)** | 58 | **5.99(4.63-7.75)** | 232 | **1.89(1.67-2.15)** | 287 | **1.15(1.03-1.29)** | 189 | **0.37(0.33-0.44)** |
| Diabetes Mellitus | 815 | **1.17(1.09-1.26)** | 77 | **10.18(8.15-12.73)** | 315 | **2.79(2.5-3.11)** | 305 | **1.16(1.04-1.3)** | 118 | **0.37(0.33-0.45)** |
| Pneumonia and Influenza | 688 | **1.1(1.02-1.19)** | 78 | **9.15(7.33-11.43)** | 284 | **2.54(2.26-2.85)** | 231 | 0.98(0.86-1.11) | 95 | **0.37(0.33-0.46)** |
| Nephritis- Nephrotic Syndrome and Nephrosis | 550 | **1.09(1-1.18)** | 49 | **7.69(5.81-10.17)** | 216 | **2.47(2.16-2.82)** | 187 | 0.98(0.85-1.13) | 98 | **0.37(0.33-0.47)** |
| Accidents and Adverse Effects | 736 | **1.21(1.12-1.3)** | 89 | **12.24(9.94-15.06)** | 329 | **3.17(2.85-3.54)** | 222 | 0.96(0.84-1.09) | 96 | **0.37(0.33-0.48)** |
| Septicemia | 410 | **1.23(1.12-1.36)** | 48 | **12.3(9.27-16.32)** | 172 | **3.09(2.66-3.58)** | 137 | 1.09(0.92-1.29) | 53 | **0.37(0.33-0.49)** |
| Hypertension without Heart Disease | 348 | **1.56(1.4-1.73)** | 31 | **10.83(7.61-15.4)** | 136 | **3.49(2.95-4.13)** | 122 | **1.45(1.21-1.73)** | 59 | **0.37(0.33-0.50)** |
| Other Infectious Diseases | 207 | **3.47(3.03-3.97)** | 23 | **35.15(23.36-52.9)** | 94 | **9.65(7.88-11.81)** | 68 | **3.01(2.37-3.81)** | 22 | **0.37(0.33-0.51)** |
| In situ- benign or unknown behavior neoplasms | 83 | **0.51(0.41-0.63)** | 10 | **5.09(2.74-9.45)** | 29 | 1.05(0.73-1.51) | 33 | **0.53(0.38-0.75)** | 11 | **0.37(0.33-0.52)** |
| Suicide and Self-Inflicted Injury | 273 | **1.67(1.49-1.88)** | 41 | **25.38(18.69-34.47)** | 117 | **4.53(3.78-5.43)** | 91 | **1.46(1.19-1.8)** | 24 | **0.37(0.33-0.53)** |
| Chronic Liver Disease and Cirrhosis | 188 | 0.94(0.81-1.08) | 17 | **9.42(5.86-15.16)** | 91 | **2.96(2.41-3.63)** | 56 | **0.73(0.56-0.95)** | 24 | **0.37(0.33-0.54)** |
| Aortic Aneurysm and Dissection | 123 | 0.84(0.7-1) | 29 | **17.71(12.3-25.48)** | 45 | **1.87(1.4-2.5)** | 40 | **0.72(0.53-0.98)** | 9 | — |
| Other Diseases of Arteries- Arterioles- Capillaries | 98 | 1.04(0.85-1.27) | 9 | — | 43 | **2.68(1.99-3.61)** | 34 | 0.95(0.68-1.33) | 12 | **0.37(0.33-0.56)** |
| Atherosclerosis | 106 | 1.16(0.96-1.4) | 13 | **10.24(5.95-17.64)** | 47 | **2.84(2.13-3.78)** | 31 | **0.9(0.63-1.27)** | 15 | **0.37(0.33-0.57)** |
| Other cancers (Non-prostate) causes of death | 1036 | **0.21(0.2-0.22)** | 165 | **3.25(2.79-3.78)** | 409 | **0.52(0.47-0.57)** | 316 | **0.17(0.15-0.19)** | 146 | **0.37(0.33-0.58)** |

Bolded SMRs are significantly different from 1.00 (P < .05). A blank indicates that because of 0 observed deaths, the SMR, 95% CI, and P value were not calculated.

**Supplementary Table 8.** Standardized-mortality ratios (SMRs) for each cause of death following prostate cancer diagnosis in single males.

| Cause of death | **Total** | | **< 1 Year** | | **1-5 Years** | | **5-10 Years** | | **≥10 Years** | |
| --- | --- | --- | --- | --- | --- | --- | --- | --- | --- | --- |
|  | Observed | SMR | Observed | SMR | Observed | SMR | Observed | SMR | Observed | SMR |
|  |  | (95% CI) |  | (95% CI) |  | (95% CI) |  | (95% CI) |  | (95% CI) |
| All causes of death | 18174 | **1.38(1.36-1.4)** | 2880 | **23.65(22.8-24.53)** | 7847 | **4.16(4.07-4.25)** | 5162 | **1.07(1.04-1.1)** | 2285 | **0.36(0.35-0.37)** |
| Non-cancer causes of death |  |  |  |  |  |  |  |  |  |  |
| Diseases of Heart | 4109 | **1.12(1.08-1.15)** | 507 | **14.71(13.48-16.05)** | 1625 | **3.08(2.94-3.24)** | 1355 | 1.01(0.96-1.06) | 622 | **0.35(0.32-0.38)** |
| Chronic Obstructive Pulmonary Disease | 811 | 0.97(0.91-1.04) | 69 | **9.32(7.36-11.8)** | 317 | **2.73(2.45-3.05)** | 279 | 0.92(0.82-1.03) | 146 | **0.36(0.31-0.42)** |
| Cerebrovascular Diseases | 686 | 1.03(0.95-1.11) | 83 | **13.29(10.72-16.48)** | 252 | **2.67(2.36-3.02)** | 239 | 0.99(0.87-1.12) | 112 | **0.34(0.29-0.41)** |
| Alzheimer’s | 323 | **1.16(1.04-1.3)** | 21 | **7.45(4.86-11.42)** | 84 | **2.13(1.72-2.63)** | 128 | **1.3(1.09-1.54)** | 90 | **0.66(0.53-0.81)** |
| Diabetes Mellitus | 476 | **1.12(1.03-1.23)** | 45 | **11.84(8.84-15.86)** | 180 | **2.97(2.57-3.44)** | 170 | 1.09(0.93-1.26) | 81 | **0.4(0.32-0.49)** |
| Pneumonia and Influenza | 366 | **1.23(1.11-1.36)** | 36 | **12.38(8.93-17.17)** | 142 | **3.33(2.83-3.93)** | 122 | 1.14(0.95-1.36) | 66 | **0.46(0.36-0.58)** |
| Nephritis- Nephrotic Syndrome and Nephrosis | 297 | **1.13(1.01-1.27)** | 34 | **13.82(9.87-19.34)** | 107 | **2.87(2.37-3.47)** | 114 | 1.19(0.99-1.43) | 42 | **0.33(0.24-0.44)** |
| Accidents and Adverse Effects | 437 | **1.15(1.05-1.27)** | 50 | **13.74(10.42-18.13)** | 191 | **3.38(2.94-3.9)** | 133 | 0.94(0.8-1.12) | 63 | **0.36(0.28-0.45)** |
| Septicemia | 214 | **1.15(1-1.31)** | 25 | **14.7(9.93-21.75)** | 96 | **3.63(2.97-4.44)** | 58 | 0.85(0.66-1.1) | 35 | **0.39(0.28-0.54)** |
| Hypertension without Heart Disease | 217 | **1.85(1.62-2.11)** | 27 | **24.13(16.55-35.19)** | 86 | **5.09(4.12-6.28)** | 67 | **1.57(1.23-1.99)** | 37 | **0.66(0.47-0.9)** |
| Other Infectious Diseases | 172 | **4.68(4.03-5.44)** | 22 | **66.27(43.64-100.65)** | 68 | **12.89(10.16-16.35)** | 55 | **4.05(3.11-5.28)** | 27 | **1.54(1.05-2.24)** |
| In situ- benign or unknown behavior neoplasms | 34 | **0.39(0.28-0.54)** | 3 | — | 17 | 1.38(0.86-2.23) | 10 | **0.31(0.17-0.58)** | 4 | — |
| Suicide and Self-Inflicted Injury | 159 | **1.27(1.09-1.49)** | 21 | **17.93(11.69-27.5)** | 73 | **3.86(3.07-4.85)** | 47 | 0.99(0.75-1.32) | 18 | **0.31(0.2-0.5)** |
| Chronic Liver Disease and Cirrhosis | 164 | 0.99(0.85-1.15) | 12 | **7.79(4.43-13.72)** | 88 | **3.47(2.82-4.28)** | 43 | **0.68(0.5-0.91)** | 21 | **0.28(0.18-0.42)** |
| Aortic Aneurysm and Dissection | 51 | **0.6(0.46-0.79)** | 7 | — | 31 | **2.6(1.83-3.7)** | 7 | — | 6 | — |
| Other Diseases of Arteries- Arterioles- Capillaries | 55 | 1.08(0.83-1.41) | 6 | — | 24 | **3.34(2.24-4.98)** | 18 | 0.97(0.61-1.54) | 7 | — |
| Atherosclerosis | 61 | **1.43(1.11-1.83)** | 5 | — | 27 | **4.42(3.03-6.44)** | 15 | 0.98(0.59-1.62) | 14 | 0.67(0.4-1.13) |
| Other cancers (Non-prostate) causes of death | 621 | **0.19(0.18-0.21)** | 96 | **3.42(2.8-4.17)** | 264 | **0.58(0.51-0.65)** | 179 | **0.15(0.13-0.17)** | 82 | **0.05(0.04-0.07)** |

Bolded SMRs are significantly different from 1.00 (P < .05). A blank indicates that because of 0 observed deaths, the SMR, 95% CI, and P value were not calculated.

**Supplementary Table 9.**  Standardized-mortality ratios (SMRs) for each cause of death following prostate cancer diagnosis in white males.

| Cause of death | **Total** | | **< 1 Year** | | **1-5 Years** | | **5-10 Years** | | **≥10 Years** | |
| --- | --- | --- | --- | --- | --- | --- | --- | --- | --- | --- |
|  | Observed | SMR | Observed | SMR | Observed | SMR | Observed | SMR | Observed | SMR |
|  |  | (95% CI) |  | (95% CI) |  | (95% CI) |  | (95% CI) |  | (95% CI) |
| All causes of death | 139025 | **0.92(0.92-0.93)** | 16214 | **16.64(16.39-16.9)** | 54147 | **2.96(2.94-2.98)** | 45430 | **0.85(0.85-0.86)** | 23234 | **0.3(0.29-0.3)** |
| Non-cancer causes of death |  |  |  |  |  |  |  |  |  |  |
| Diseases of Heart | 34178 | **0.8(0.79-0.81)** | 3191 | **11.26(10.88-11.66)** | 12485 | **2.38(2.34-2.43)** | 12119 | **0.8(0.79-0.82)** | 6383 | **0.29(0.28-0.3)** |
| Chronic Obstructive Pulmonary Disease | 7478 | **0.77(0.75-0.79)** | 562 | **9.27(8.53-10.07)** | 2760 | **2.38(2.29-2.47)** | 2756 | **0.8(0.78-0.84)** | 1400 | **0.28(0.26-0.29)** |
| Cerebrovascular Diseases | 6312 | **0.78(0.77-0.8)** | 545 | **10.2(9.38-11.1)** | 2198 | **2.24(2.15-2.33)** | 2315 | **0.82(0.79-0.85)** | 1254 | **0.3(0.28-0.32)** |
| Alzheimer’s | 4408 | **1.16(1.13-1.2)** | 173 | **6.24(5.38-7.25)** | 1152 | **2.42(2.29-2.57)** | 1792 | **1.36(1.3-1.42)** | 1291 | **0.66(0.62-0.69)** |
| Diabetes Mellitus | 3288 | **0.71(0.69-0.74)** | 275 | **9.62(8.55-10.83)** | **1184** | **2.13(2.02-2.26)** | 1224 | **0.75(0.71-0.79)** | 605 | **0.25(0.23-0.27)** |
| Pneumonia and Influenza | 2829 | **0.76(0.73-0.79)** | 241 | **9.21(8.11-10.45)** | **973** | **2.09(1.97-2.23)** | 1051 | **0.8(0.76-0.85)** | 564 | **0.29(0.27-0.32)** |
| Nephritis- Nephrotic Syndrome and Nephrosis | 2129 | **0.68(0.65-0.71)** | 141 | **6.73(5.71-7.94)** | **689** | **1.79(1.66-1.93)** | 832 | **0.75(0.7-0.8)** | 467 | **0.29(0.26-0.31)** |
| Accidents and Adverse Effects | 3519 | **0.89(0.86-0.92)** | 314 | **11.97(10.72-13.37)** | **1355** | **2.76(2.62-2.91)** | 1225 | **0.87(0.82-0.92)** | 625 | **0.31(0.28-0.33)** |
| Septicemia | 1438 | **0.67(0.64-0.71)** | 126 | **9.23(7.75-10.99)** | **558** | **2.16(1.99-2.35)** | 496 | **0.66(0.6-0.72)** | 258 | **0.23(0.21-0.26)** |
| Hypertension without Heart Disease | 1321 | 0.96(0.91-1.01) | 93 | **9.93(8.1-12.17)** | **444** | **2.6(2.37-2.86)** | 499 | 1.02(0.94-1.12) | 285 | **0.4(0.36-0.45)** |
| Other Infectious Diseases | 798 | **2.01(1.88-2.16)** | 74 | **29.96(23.86-37.63)** | 314 | **6.57(5.88-7.34)** | 264 | **1.88(1.66-2.12)** | 146 | **0.71(0.6-0.84)** |
| In situ- benign or unknown behavior neoplasms | 419 | **0.4(0.37-0.44)** | 36 | **5.39(3.89-7.48)** | 136 | **1.09(0.92-1.29)** | 162 | **0.44(0.38-0.52)** | 85 | **0.16(0.13-0.19)** |
| Suicide and Self-Inflicted Injury | 1275 | **1.11(1.05-1.17)** | 168 | **23.6(20.29-27.45)** | 505 | **3.58(3.28-3.9)** | 441 | 1.07(0.97-1.17) | 161 | **0.27(0.24-0.32)** |
| Chronic Liver Disease and Cirrhosis | 947 | **0.65(0.61-0.69)** | 72 | **8.12(6.45-10.24)** | 441 | **2.45(2.23-2.69)** | 286 | **0.54(0.48-0.6)** | 148 | **0.2(0.17-0.23)** |
| Aortic Aneurysm and Dissection | 634 | **0.66(0.61-0.71)** | 67 | **11.24(8.85-14.29)** | 267 | **2.33(2.06-2.62)** | 216 | **0.64(0.56-0.73)** | 84 | **0.17(0.14-0.21)** |
| Other Diseases of Arteries- Arterioles- Capillaries | 413 | **0.69(0.63-0.76)** | 29 | **7.46(5.19-10.74)** | 162 | **2.24(1.92-2.61)** | 135 | **0.64(0.54-0.76)** | 87 | **0.28(0.23-0.35)** |
| Atherosclerosis | 449 | **0.83(0.75-0.91)** | 43 | **11.18(8.29-15.08)** | 158 | **2.33(1.99-2.72)** | 165 | 0.87(0.74-1.01) | 83 | **0.29(0.24-0.37)** |
| Other cancers (Non-prostate) causes of death | 4413 | **0.13(0.13-0.13)** | 534 | **2.62(2.4-2.85)** | 1575 | **0.39(0.37-0.41)** | 1456 | **0.12(0.11-0.13)** | 848 | **0.05(0.04-0.05)** |

Bolded SMRs are significantly different from 1.00 (P < .05). A blank indicates that because of 0 observed deaths, the SMR, 95% CI, and P value were not calculated.

**Supplementary Table 10.**  Standardized-mortality ratios (SMRs) for each cause of death following prostate cancer diagnosis in black males.

| Cause of death | **Total** | | **< 1 Year** | | **1-5 Years** | | **5-10 Years** | | **≥10 Years** | |
| --- | --- | --- | --- | --- | --- | --- | --- | --- | --- | --- |
|  | Observed | SMR | Observed | SMR | Observed | SMR | Observed | SMR | Observed | SMR |
|  |  | (95% CI) |  | (95% CI) |  | (95% CI) |  | (95% CI) |  | (95% CI) |
| All causes of death | 28545 | **1.34(1.33-1.36)** | 3952 | **23.53(22.81-24.27)** | 12039 | **4.06(3.99-4.13)** | 8659 | **1.11(1.08-1.13)** | 3895 | **0.38(0.37-0.39)** |
| Non-cancer causes of death |  |  |  |  |  |  |  |  |  |  |
| Diseases of Heart | 6901 | **1.17(1.14-1.19)** | 768 | **16.2(15.09-17.38)** | 2753 | **3.33(3.21-3.45)** | 2331 | **1.07(1.03-1.12)** | 1049 | **0.37(0.34-0.39)** |
| Chronic Obstructive Pulmonary Disease | 1060 | **0.78(0.74-0.83)** | 97 | **9.43(7.73-11.51)** | 401 | **2.18(1.97-2.4)** | 394 | **0.8(0.72-0.88)** | 168 | **0.25(0.22-0.29)** |
| Cerebrovascular Diseases | 1344 | **1.25(1.18-1.32)** | 139 | **16.18(13.7-19.1)** | 528 | **3.54(3.25-3.86)** | 466 | **1.18(1.08-1.3)** | 211 | **0.4(0.35-0.46)** |
| Alzheimer’s | 523 | **1.17(1.08-1.28)** | 29 | **7.57(5.26-10.89)** | 127 | **2.05(1.72-2.44)** | 210 | **1.3(1.14-1.49)** | 157 | **0.72(0.61-0.84)** |
| Diabetes Mellitus | 1064 | **1.55(1.46-1.65)** | 104 | **19.73(16.28-23.91)** | 435 | **4.56(4.15-5.01)** | 351 | **1.39(1.25-1.54)** | 174 | **0.52(0.45-0.61)** |
| Pneumonia and Influenza | 488 | **1.02(0.93-1.11)** | 45 | **11.32(8.45-15.16)** | 172 | **2.57(2.21-2.99)** | 191 | 1.09(0.95-1.26) | 80 | **0.34(0.28-0.43)** |
| Nephritis- Nephrotic Syndrome and Nephrosis | 779 | **1.84(1.71-1.97)** | 79 | **23.33(18.71-29.08)** | 294 | **5(4.46-5.61)** | 259 | **1.67(1.48-1.88)** | 147 | **0.71(0.6-0.83)** |
| Accidents and Adverse Effects | 517 | **0.86(0.79-0.93)** | 61 | **12.28(9.55-15.78)** | 245 | **2.8(2.47-3.18)** | 151 | **0.67(0.57-0.79)** | 60 | **0.21(0.16-0.27)** |
| Septicemia | 550 | **1.83(1.68-1.99)** | 71 | **30.21(23.94-38.12)** | 215 | **5.16(4.51-5.9)** | 188 | **1.7(1.48-1.96)** | 76 | **0.52(0.41-0.65)** |
| Hypertension without Heart Disease | 425 | **2.25(2.05-2.48)** | 37 | **24.1(17.46-33.27)** | 165 | **6.23(5.35-7.26)** | 142 | **2.05(1.74-2.42)** | 81 | **0.89(0.71-1.1)** |
| Other Infectious Diseases | 222 | **3.75(3.28-4.27)** | 31 | **67.49(47.46-95.97)** | 96 | **11.59(9.48-14.15)** | 75 | **3.43(2.74-4.3)** | 20 | **0.7(0.45-1.08)** |
| In situ- benign or unknown behavior neoplasms | 57 | **0.4(0.31-0.52)** | 6 | — | 23 | 1.18(0.79-1.78) | 20 | **0.39(0.25-0.6)** | 8 | — |
| Suicide and Self-Inflicted Injury | 88 | **0.44(0.36-0.55)** | 14 | **8.74(5.18-14.76)** | 33 | 1.13(0.8-1.59) | 29 | **0.39(0.27-0.56)** | 12 | **0.13(0.07-0.23)** |
| Chronic Liver Disease and Cirrhosis | 129 | **0.49(0.41-0.58)** | 13 | **6.15(3.57-10.59)** | 61 | **1.57(1.22-2.01)** | 33 | **0.33(0.24-0.47)** | 22 | **0.18(0.12-0.27)** |
| Aortic Aneurysm and Dissection | 101 | **0.74(0.6-0.89)** | 17 | **16.23(10.09-26.11)** | 49 | **2.6(1.96-3.44)** | 27 | **0.54(0.37-0.78)** | 8 | — |
| Other Diseases of Arteries- Arterioles- Capillaries | 122 | **1.49(1.24-1.77)** | 12 | **18.6(10.57-32.76)** | 50 | **4.41(3.34-5.82)** | 38 | **1.26(0.92-1.74)** | 22 | **0.55(0.36-0.83)** |
| Atherosclerosis | 82 | 1.19(0.96-1.48) | 6 | — | 30 | **3.13(2.19-4.47)** | 28 | **1.12(0.77-1.62)** | 18 | **0.54(0.34-0.85)** |
| Other cancers (Non-prostate) causes of death | 938 | **0.18(0.17-0.19)** | 114 | **2.91(2.43-3.5)** | 352 | **0.49(0.44-0.54)** | 323 | **0.17(0.15-0.19)** | 149 | **0.06(0.05-0.07)** |

Bolded SMRs are significantly different from 1.00 (P < .05). A blank indicates that because of 0 observed deaths, the SMR, 95% CI, and P value were not calculated.

**Supplementary Table 11.** Standardized-mortality ratios (SMRs) for each cause of death following prostate cancer diagnosis in American india/Alaska Native Ssian or Pacific Islander males.

| Cause of death | **Total** | | **< 1 Year** | | **1-5 Years** | | **5-10 Years** | | **≥10 Years** | |
| --- | --- | --- | --- | --- | --- | --- | --- | --- | --- | --- |
|  | Observed | SMR | Observed | SMR | Observed | SMR | Observed | SMR | Observed | SMR |
|  |  | (95% CI) |  | (95% CI) |  | (95% CI) |  | (95% CI) |  | (95% CI) |
| All causes of death | 8690 | **0.77(0.76-0.79)** | 972 | **13.26(12.45-14.12)** | 3477 | **2.47(2.39-2.55)** | 2781 | **0.7(0.67-0.72)** | 1460 | **0.25(0.24-0.27)** |
| Non-cancer causes of death |  |  |  |  |  |  |  |  |  |  |
| Diseases of Heart | 1871 | **0.58(0.55-0.61)** | 179 | **8.48(7.33-9.82)** | 716 | **1.77(1.64-1.9)** | 625 | **0.55(0.5-0.59)** | 351 | **0.21(0.19-0.24)** |
| Chronic Obstructive Pulmonary Disease | 364 | **0.5(0.45-0.55)** | 29 | **6.25(4.34-9)** | 148 | **1.64(1.4-1.93)** | 114 | **0.44(0.37-0.53)** | 73 | **0.2(0.16-0.25)** |
| Cerebrovascular Diseases | 508 | **0.82(0.75-0.9)** | 43 | **10.82(8.02-14.58)** | 179 | **2.34(2.02-2.7)** | 179 | **0.82(0.71-0.95)** | 107 | **0.33(0.28-0.4)** |
| Alzheimer’s | 178 | **0.57(0.49-0.66)** | 7 | — | 43 | **1.13(0.84-1.53)** | 67 | **0.62(0.49-0.79)** | 61 | **0.37(0.29-0.48)** |
| Diabetes Mellitus | 243 | **0.73(0.64-0.83)** | 20 | **9.11(5.88-14.12)** | 97 | **2.29(1.88-2.8)** | 86 | **0.71(0.58-0.88)** | 40 | **0.24(0.17-0.32)** |
| Pneumonia and Influenza | 303 | 1.03(0.92-1.15) | 15 | **7.88(4.75-13.06)** | 98 | **2.68(2.2-3.27)** | 121 | 1.17(0.98-1.4) | 69 | **0.45(0.36-0.57)** |
| Nephritis- Nephrotic Syndrome and Nephrosis | 130 | **0.54(0.45-0.64)** | 6 | — | 37 | **1.23(0.89-1.7)** | 59 | **0.69(0.54-0.89)** | 28 | **0.22(0.16-0.33)** |
| Accidents and Adverse Effects | 209 | **0.73(0.64-0.84)** | 14 | **7.21(4.27-12.17)** | 75 | **2.03(1.62-2.55)** | 72 | **0.7(0.56-0.88)** | 48 | **0.33(0.25-0.44)** |
| Septicemia | 85 | **0.53(0.43-0.66)** | 9 | — | 30 | **1.51(1.05-2.16)** | 32 | **0.56(0.4-0.8)** | 14 | **0.17(0.1-0.29)** |
| Hypertension without Heart Disease | 94 | 0.89(0.73-1.09) | 6 | — | 32 | **2.42(1.71-3.42)** | 38 | **1.02(0.74-1.4)** | 18 | **0.33(0.21-0.53)** |
| Other Infectious Diseases | 55 | **1.92(1.48-2.51)** | 8 | — | 24 | **6.61(4.43-9.86)** | 16 | 1.55(0.95-2.53) | 7 | — |
| In situ- benign or unknown behavior neoplasms | 22 | **0.28(0.18-0.42)** | 0 | — | 8 | — | 10 | **0.36(0.19-0.66)** | 4 | — |
| Suicide and Self-Inflicted Injury | 38 | **0.5(0.36-0.68)** | 3 | — | 20 | **1.96(1.26-3.04)** | 13 | **0.46(0.27-0.79)** | 2 | — |
| Chronic Liver Disease and Cirrhosis | 36 | **0.39(0.28-0.53)** | 2 | — | 18 | 1.4(0.89-2.23) | 11 | **0.31(0.17-0.57)** | 5 | — |
| Aortic Aneurysm and Dissection | 46 | **0.65(0.49-0.87)** | 9 | — | 13 | 1.47(0.85-2.53) | 14 | **0.55(0.33-0.93)** | 10 | **0.28(0.15-0.51)** |
| Other Diseases of Arteries- Arterioles- Capillaries | 11 |  | 0 | — | 3 | — | 7 | — | 1 | — |
| Atherosclerosis | 16 | **0.37(0.23-0.6)** | 2 | — | 7 | — | 4 | — | 3 | — |
| Other cancers (Non-prostate) causes of death | 205 | **0.09(0.07-0.1)** | 35 | **2.2(1.58-3.07)** | 71 | **0.23(0.18-0.29)** | 69 | **0.08(0.06-0.1)** | 30 | **0.02(0.02-0.04)** |

Bolded SMRs are significantly different from 1.00 (P < .05). A blank indicates that because of 0 observed deaths, the SMR, 95% CI, and P value were not calculated.

**Supplementary Table 12.** Standardized-mortality ratios (SMRs) for each cause of death following local prostate cancer diagnosis.

| Cause of death | **Total** | | **< 1 Year** | | **1-5 Years** | | **5-10 Years** | | **≥10 Years** | |
| --- | --- | --- | --- | --- | --- | --- | --- | --- | --- | --- |
|  | Observed | SMR | Observed | SMR | Observed | SMR | Observed | SMR | Observed | SMR |
|  |  | (95% CI) |  | (95% CI) |  | (95% CI) |  | (95% CI) |  | (95% CI) |
| All causes of death | 122536 | **0.78(0.77-0.78)** | 8472 | **12.78(12.51-13.06)** | 43603 | **2.52(2.5-2.55)** | 46260 | **0.82(0.81-0.82)** | 24201 | **0.29(0.29-0.3)** |
| Non-cancer causes of death |  |  |  |  |  |  |  |  |  |  |
| Diseases of Heart | 35726 | **0.8(0.79-0.81)** | 2856 | **15.28(14.73-15.85)** | 12830 | **2.62(2.58-2.67)** | 13192 | **0.82(0.81-0.84)** | 6848 | **0.29(0.28-0.3)** |
| Chronic Obstructive Pulmonary Disease | 7524 | **0.74(0.72-0.76)** | 480 | **11.52(10.53-12.6)** | 2741 | **2.49(2.4-2.59)** | 2888 | **0.79(0.76-0.82)** | 1415 | **0.26(0.25-0.28)** |
| Cerebrovascular Diseases | 6872 | **0.82(0.8-0.83)** | 502 | **14.65(13.42-15.99)** | 2376 | **2.61(2.5-2.71)** | 2600 | **0.86(0.83-0.9)** | 1394 | **0.31(0.3-0.33)** |
| Alzheimer’s | 4355 | **1.1(1.07-1.13)** | 124 | **8.11(6.8-9.67)** | 1066 | **2.53(2.38-2.69)** | 1844 | **1.31(1.26-1.38)** | 1321 | **0.62(0.59-0.66)** |
| Diabetes Mellitus | 3873 | **0.8(0.78-0.83)** | 288 | **13.84(12.33-15)** | 1431 | **2.68(2.54-2.82)** | 1452 | **0.83(0.79-0.87)** | 702 | **0.28(0.26-0.3)** |
| Pneumonia and Influenza | 3010 | **0.77(0.74-0.8)** | 178 | **11.3(9.75-13.08)** | 1015 | **2.4(2.26-2.55)** | 1187 | **0.85(0.8-0.9)** | 630 | **0.3(0.28-0.33)** |
| Nephritis- Nephrotic Syndrome and Nephrosis | 2577 | **0.78(0.75-0.81)** | 159 | **11.79(10.09-13.77)** | 830 | **2.32(2.17-2.48)** | 1022 | **0.87(0.81-0.92)** | 566 | **0.32(0.3-0.35)** |
| Accidents and Adverse Effects | 3549 | **0.86(0.83-0.88)** | 273 | **14.71(13.07-16.57)** | 1362 | **2.91(2.76-3.07)** | 1278 | **0.85(0.8-0.9)** | 636 | **0.29(0.27-0.32)** |
| Septicemia | 1670 | **0.75(0.71-0.78)** | 129 | **13.82(11.63-16.43)** | 608 | **2.49(2.3-2.7)** | 622 | **0.77(0.72-0.84)** | 311 | **0.26(0.24-0.3)** |
| Hypertension without Heart Disease | 1537 | **1.06(1.01-1.12)** | 78 | **12.93(10.36-16.15)** | 516 | **3.26(2.99-3.55)** | 601 | **1.16(1.07-1.26)** | 342 | **0.45(0.4-0.5)** |
| Other Infectious Diseases | 881 | **2.12(1.99-2.27)** | 72 | **40.05(31.79-50.45)** | 342 | **7.43(6.68-8.26)** | 315 | **2.1(1.88-2.34)** | 152 | **0.7(0.6-0.82)** |
| In situ- benign or unknown behavior neoplasms | 411 | **0.38(0.34-0.42)** | 26 | **5.87(4-8.62)** | 133 | **1.13(0.96-1.34)** | 166 | **0.43(0.37-0.5)** | 86 | **0.15(0.12-0.18)** |
| Suicide and Self-Inflicted Injury | 1125 | **0.93(0.88-0.99)** | 136 | **23.4(19.78-27.68)** | 423 | **3.01(2.74-3.31)** | 415 | **0.94(0.85-1.03)** | 151 | **0.25(0.21-0.29)** |
| Chronic Liver Disease and Cirrhosis | 903 | **0.59(0.55-0.63)** | 60 | **7.82(6.07-10.07)** | 415 | **2.28(2.07-2.51)** | 282 | **0.5(0.44-0.56)** | 146 | **0.19(0.16-0.22)** |
| Aortic Aneurysm and Dissection | 664 | **0.66(0.61-0.71)** | 76 | **18.11(14.46-22.67)** | 272 | **2.48(2.2-2.79)** | 225 | **0.62(0.55-0.71)** | 91 | **0.17(0.14-0.21)** |
| Other Diseases of Arteries- Arterioles- Capillaries | 457 | **0.73(0.67-0.8)** | 28 | **10.86(7.5-15.72)** | 175 | **2.58(2.22-2.99)** | 162 | **0.72(0.62-0.84)** | 92 | **0.28(0.23-0.34)** |
| Atherosclerosis | 443 | **0.78(0.71-0.85)** | 29 | **12.72(8.84-18.31)** | 153 | **2.49(2.13-2.92)** | 166 | **0.82(0.7-0.95)** | 95 | **0.31(0.26-0.38)** |
| Other cancers (Non-prostate) causes of death | 3860 | **0.11(0.1-0.11)** | 192 | **1.24(1.08-1.43)** | 1298 | **0.33(0.31-0.35)** | 1528 | **0.12(0.11-0.12)** | 842 | **0.05(0.04-0.05)** |

Bolded SMRs are significantly different from 1.00 (P < .05). A blank indicates that because of 0 observed deaths, the SMR, 95% CI, and P value were not calculated.

**Supplementary Table 13.**  Standardized-mortality ratios (SMRs) for each cause of death following regional prostate cancer diagnosis.

| Cause of death | **Total** | | **< 1 Year** | | **1-5 Years** | | **5-10 Years** | | **≥10 Years** | |
| --- | --- | --- | --- | --- | --- | --- | --- | --- | --- | --- |
|  | Observed | SMR | Observed | SMR | Observed | SMR | Observed | SMR | Observed | SMR |
|  |  | (95% CI) |  | (95% CI) |  | (95% CI) |  | (95% CI) |  | (95% CI) |
| All causes of death | 14923 | **0.89(0.87-0.9)** | 1020 | **10.04(9.44-10.67)** | 5571 | **2.91(2.83-2.99)** | 5373 | 0.99(0.96-1.02) | 2959 | **0.31(0.3-0.33)** |
| Non-cancer causes of death |  |  |  |  |  |  |  |  |  |  |
| Diseases of Heart | 2797 | **0.61(0.59-0.63)** | 212 | **7.51(6.56-8.59)** | 1016 | **1.94(1.82-2.06)** | 948 | **0.64(0.6-0.69)** | 621 | **0.24(0.22-0.26)** |
| Chronic Obstructive Pulmonary Disease | 513 | **0.48(0.44-0.52)** | 24 | **3.91(2.62-5.83)** | 140 | **1.19(1.01-1.41)** | 198 | **0.58(0.5-0.67)** | 151 | **0.25(0.21-0.29)** |
| Cerebrovascular Diseases | 510 | **0.63(0.58-0.69)** | 35 | **7.08(5.08-9.86)** | 158 | **1.74(1.49-2.03)** | 182 | **0.71(0.62-0.83)** | 135 | **0.29(0.25-0.35)** |
| Alzheimer’s | 290 | 1.03(0.91-1.15) | 3 | 1.53(0.49-4.75) | 57 | **1.74(1.34-2.26)** | 102 | **1.22(1-1.48)** | 128 | **0.78(0.65-0.93)** |
| Diabetes Mellitus | 320 | **0.56(0.5-0.63)** | 19 | **5.71(3.64-8.96)** | 97 | **1.51(1.24-1.84)** | 117 | **0.63(0.52-0.75)** | 87 | **0.28(0.22-0.34)** |
| Pneumonia and Influenza | 204 | **0.6(0.52-0.69)** | 12 | **5.43(3.08-9.56)** | 59 | **1.5(1.16-1.94)** | 92 | 0.87(0.71-1.07) | 41 | **0.21(0.16-0.29)** |
| Nephritis- Nephrotic Syndrome and Nephrosis | 166 | **0.52(0.44-0.6)** | 11 | **5.6(3.1-10.1)** | 51 | **1.41(1.07-1.85)** | 55 | **0.54(0.42-0.71)** | 49 | **0.27(0.2-0.36)** |
| Accidents and Adverse Effects | 347 | **0.71(0.64-0.79)** | 21 | **6.79(4.43-10.42)** | 144 | **2.48(2.11-2.92)** | 113 | **0.7(0.58-0.84)** | 69 | **0.26(0.21-0.33)** |
| Septicemia | 124 | **0.52(0.44-0.62)** | 10 | **7.05(3.79-13.1)** | 47 | **1.75(1.32-2.33)** | 45 | **0.59(0.44-0.79)** | 22 | **0.16(0.11-0.25)** |
| Hypertension without Heart Disease | 129 | 0.9(0.76-1.07) | 14 | **15.46(9.16-26.11)** | 42 | **2.53(1.87-3.42)** | 43 | 0.94(0.69-1.26) | 30 | **0.37(0.26-0.54)** |
| Other Infectious Diseases | 93 | **1.89(1.55-2.32)** | 10 | **34.54(18.59-64.2)** | 40 | **7.16(5.25-9.77)** | 28 | **1.74(1.2-2.52)** | 15 | **0.55(0.33-0.92)** |
| In situ- benign or unknown behavior neoplasms | 32 | **0.29(0.21-0.41)** | 1 | — | 7 | — | 17 | **0.49(0.31-0.79)** | 7 | — |
| Suicide and Self-Inflicted Injury | 161 | 0.93(0.8-1.08) | 12 | **11.19(6.36-19.71)** | 75 | **3.6(2.87-4.51)** | 53 | 0.89(0.68-1.17) | 21 | **0.23(0.15-0.35)** |
| Chronic Liver Disease and Cirrhosis | 127 | **0.53(0.44-0.63)** | 5 | — | 55 | **1.88(1.45-2.45)** | 41 | **0.49(0.36-0.66)** | 26 | **0.2(0.14-0.3)** |
| Aortic Aneurysm and Dissection | 67 | **0.6(0.47-0.77)** | 1 | — | 29 | **2.36(1.64-3.4)** | 27 | 0.76(0.52-1.1) | 10 | **0.16(0.09-0.3)** |
| Other Diseases of Arteries- Arterioles- Capillaries | 38 | **0.6(0.43-0.82)** | 3 | — | 11 | **1.53(0.85-2.77)** | 11 | **0.54(0.3-0.98)** | 13 | **0.36(0.21-0.62)** |
| Atherosclerosis | 27 | **0.56(0.38-0.82)** | 1 | — | 7 | — | 14 | 0.94(0.56-1.59) | 5 | — |
| Other cancers (Non-prostate) causes of death | 611 | **0.14(0.13-0.15)** | 47 | **1.86(1.4-2.47)** | 203 | **0.41(0.36-0.47)** | 213 | **0.15(0.13-0.17)** | 148 | **0.06(0.05-0.07)** |

Bolded SMRs are significantly different from 1.00 (P < .05). A blank indicates that because of 0 observed deaths, the SMR, 95% CI, and P value were not calculated.

**Supplementary Table 14.**Standardized-mortality ratios (SMRs) for each cause of death following distant prostate cancer diagnosis.

| Cause of death | **Total** | | **< 1 Year** | | **1-5 Years** | | **5-10 Years** | | **≥10 Years** | |
| --- | --- | --- | --- | --- | --- | --- | --- | --- | --- | --- |
|  | Observed | SMR | Observed | SMR | Observed | SMR | Observed | SMR | Observed | SMR |
|  |  | (95% CI) |  | (95% CI) |  | (95% CI) |  | (95% CI) |  | (95% CI) |
| All causes of death | 26426 | **5.9(5.83-5.98)** | 8804 | **27.6(27.03-28.18)** | 14812 | **6.69(6.59-6.8)** | 2408 | **1.88(1.81-1.96)** | 402 | **0.6(0.55-0.66)** |
| Non-cancer causes of death |  |  |  |  |  |  |  |  |  |  |
| Diseases of Heart | 1879 | **1.43(1.37-1.5)** | 617 | **6.46(5.97-6.99)** | 947 | **1.45(1.36-1.55)** | 250 | **0.67(0.59-0.76)** | 65 | **0.33(0.26-0.43)** |
| Chronic Obstructive Pulmonary Disease | 341 | **1.21(1.09-1.34)** | 98 | **4.95(4.06-6.03)** | 177 | **1.28(1.1-1.48)** | 52 | **0.64(0.49-0.84)** | 14 | **0.33(0.19-0.55)** |
| Cerebrovascular Diseases | 329 | **1.3(1.17-1.45)** | 100 | **5.38(4.42-6.55)** | 174 | **1.39(1.2-1.61)** | 48 | **0.67(0.51-0.89)** | 7 | —**)** |
| Alzheimer’s | 128 | 0.93(0.78-1.11) | 28 | **2.58(1.78-3.74)** | 67 | **0.97(0.76-1.23)** | 21 | **0.56(0.36-0.85)** | 12 | **0.61(0.35-1.07)** |
| Diabetes Mellitus | 165 | **1.29(1.11-1.5)** | 49 | **5.64(4.26-7.46)** | 84 | **1.34(1.08-1.66)** | 27 | 0.73(0.5-1.06) | 5 | — |
| Pneumonia and Influenza | 177 | **1.41(1.22-1.63)** | 69 | **7.21(5.69-9.13)** | 79 | **1.26(1.01-1.57)** | 22 | **0.63(0.41-0.95)** | 7 | — |
| Nephritis- Nephrotic Syndrome and Nephrosis | 119 | **1.21(1.01-1.44)** | 36 | **4.97(3.59-6.89)** | 55 | 1.12(0.86-1.46) | 22 | 0.79(0.52-1.2) | 6 | — |
| Accidents and Adverse Effects | 167 | **1.43(1.23-1.67)** | 63 | **7.67(5.99-9.82)** | 86 | **1.48(1.2-1.83)** | 15 | **0.45(0.27-0.75)** | 3 | — |
| Septicemia | 161 | **2.56(2.19-2.99)** | 48 | **10.76(8.11-14.27)** | 89 | **2.87(2.33-3.53)** | 20 | 1.11(0.72-1.72) | 4 | — |
| Hypertension without Heart Disease | 81 | **1.86(1.49-2.31)** | 28 | **8.69(6-12.58)** | 37 | **1.7(1.23-2.35)** | 11 | **0.89(0.5-1.62)** | 5 | — |
| Other Infectious Diseases | 50 | **4.54(3.44-5.99)** | 17 | **22.66(14.09-36.45)** | 25 | **4.62(3.12-6.84)** | 7 | — | 1 | — |
| In situ- benign or unknown behavior neoplasms | 26 | 0.83(0.56-1.22) | 7 | — | 14 | 0.9(0.53-1.53) | 4 | — | 1 | — |
| Suicide and Self-Inflicted Injury | 65 | **2.23(1.75-2.84)** | 22 | **11.94(7.86-18.13)** | 38 | **2.64(1.92-3.62)** | 5 | — | 0 | — |
| Chronic Liver Disease and Cirrhosis | 45 | **1.31(0.98-1.76)** | 17 | **8.35(5.19-13.43)** | 26 | **1.54(1.05-2.27)** | 2 | — | 0 | — |
| Aortic Aneurysm and Dissection | 21 | 0.77(0.5-1.18) | 5 | — | 16 | 1.19(0.73-1.95) | 0 | — | 0 | — |
| Other Diseases of Arteries- Arterioles- Capillaries | 21 | 1.16(0.76-1.78) | 8 | — | 9 | — | 1 | — | 3 | — |
| Atherosclerosis | 29 | **1.57(1.09-2.25)** | 9 | — | 16 | **1.73(1.06-2.82)** | 4 | — | 0 | — |
| Other cancers (Non-prostate) causes of death | 804 | **0.9(0.84-0.96)** | 373 | **6.34(5.73-7.02)** | 358 | **0.82(0.74-0.91)** | 63 | **0.24(0.19-0.31)** | 10 | **0.07(0.04-0.14)** |

Bolded SMRs are significantly different from 1.00 (P < .05). A blank indicates that because of 0 observed deaths, the SMR, 95% CI, and P value were not calculated.

**Supplementary Table 15.** Standardized-mortality ratios (SMRs) for each cause of death following prostate cancer diagnosis in males with grade I

| Cause of death | **Total** | | **< 1 Year** | | **1-5 Years** | | **5-10 Years** | | **≥10 Years** | |
| --- | --- | --- | --- | --- | --- | --- | --- | --- | --- | --- |
|  | Observed | SMR | Observed | SMR | Observed | SMR | Observed | SMR | Observed | SMR |
|  |  | (95% CI) |  | (95% CI) |  | (95% CI) |  | (95% CI) |  | (95% CI) |
| All causes of death | 3616 | **0.75(0.73-0.77)** | 425 | **3.14(2.85-3.45)** | 1212 | **0.91(0.86-0.96)** | 1076 | **1.22(1.14-1.29)** | 903 | **0.36(0.34-0.39)** |
| Non-cancer causes of death |  |  |  |  |  |  |  |  |  |  |
| Diseases of Heart | 1209 | **0.88(0.84-0.94)** | 141 | **3.86(3.28-4.56)** | 435 | **1.2(1.09-1.32)** | 366 | **1.43(1.29-1.59)** | 267 | **0.37(0.33-0.42)** |
| Chronic Obstructive Pulmonary Disease | 245 | **0.79(0.7-0.9)** | 22 | **2.65(1.75-4.03)** | 90 | 1.08(0.88-1.33) | 72 | **1.26(1-1.58)** | 61 | **0.38(0.29-0.49)** |
| Cerebrovascular Diseases | 204 | **0.8(0.7-0.92)** | 21 | **3.39(2.21-5.19)** | 56 | 0.88(0.68-1.15) | 79 | **1.61(1.29-2.01)** | 48 | **0.35(0.26-0.47)** |
| Alzheimer’s | 153 | **1.29(1.1-1.52)** | 7 | **3.62(1.72-7.59)** | 32 | **1.43(1.01-2.02)** | 50 | **2.02(1.53-2.66)** | 64 | **0.93(0.72-1.18)** |
| Diabetes Mellitus | 126 | 0.84(0.71-1.01) | 16 | **3.41(2.09-5.57)** | 46 | 1.02(0.77-1.37) | 35 | 1.33(0.96-1.86) | 29 | **0.39(0.27-0.57)** |
| Pneumonia and Influenza | 104 | 0.88(0.73-1.07) | 15 | **5.86(3.53-9.72)** | 34 | 1.26(0.9-1.76) | 37 | **1.58(1.15-2.18)** | 18 | **0.28(0.17-0.44)** |
| Nephritis- Nephrotic Syndrome and Nephrosis | 95 | 0.95(0.78-1.16) | 9 | — | 41 | **1.62(1.2-2.21)** | 18 | 0.94(0.59-1.5) | 27 | **0.51(0.35-0.74)** |
| Accidents and Adverse Effects | 102 | **0.79(0.65-0.95)** | 19 | **4.5(2.87-7.05)** | 40 | 1(0.74-1.37) | 18 | 0.79(0.5-1.25) | 25 | **0.4(0.27-0.59)** |
| Septicemia | 50 | **0.73(0.55-0.97)** | 5 | — | 22 | 1.17(0.77-1.78) | 14 | 1.12(0.66-1.88) | 9 | —**)** |
| Hypertension without Heart Disease | 55 | 1.25(0.96-1.62) | 7 | — | 14 | 1.22(0.73-2.07) | 18 | **2.16(1.36-3.43)** | 16 | 0.69(0.42-1.12) |
| Other Infectious Diseases | 29 | **2.26(1.57-3.25)** | 6 | — | 11 | **2.83(1.56-5.1)** | 8 | — | 4 | — |
| In situ- benign or unknown behavior neoplasms | 17 | **0.52(0.32-0.83)** | 1 | — | 6 | — | 7 | 1.13(0.54-2.36) | 3 | — |
| Suicide and Self-Inflicted Injury | 39 | 1.01(0.74-1.38) | 8 | — | 12 | 0.84(0.47-1.47) | 13 | **2.14(1.24-3.69)** | 6 | — |
| Chronic Liver Disease and Cirrhosis | 32 | **0.64(0.45-0.91)** | 6 | — | 12 | 0.6(0.34-1.06) | 6 | **0.81(0.37-1.81)** | 8 | — |
| Aortic Aneurysm and Dissection | 22 | 0.72(0.47-1.09) | 2 | — | 9 | — | 7 | 1.26(0.6-2.63) | 4 | — |
| Other Diseases of Arteries- Arterioles- Capillaries | 13 | 0.68(0.4-1.18) | 1 | — | 10 | **2(1.08-3.72)** | 1 | 0.28(0.04-1.99) | 1 | — |
| Atherosclerosis | 14 | 0.82(0.48-1.38) | 2 | — | 4 | — | 5 | 1.46(0.61-3.5) | 3 | — |
| Other cancers (Non-prostate) causes of death | 113 | **0.1(0.09-0.12)** | 11 | **0.3(0.17-0.54)** | 37 | **0.11(0.08-0.15)** | 35 | **0.19(0.13-0.26)** | 30 | **0.06(0.04-0.08)** |

Bolded SMRs are significantly different from 1.00 (P < .05). A blank indicates that because of 0 observed deaths, the SMR, 95% CI, and P value were not calculated.

**Supplementary Table 16.**Standardized-mortality ratios (SMRs) for each cause of death following prostate cancer diagnosis in males with grade II

| Cause of death | **Total** | | **< 1 Year** | | **1-5 Years** | | **5-10 Years** | | **≥10 Years** | |
| --- | --- | --- | --- | --- | --- | --- | --- | --- | --- | --- |
|  | Observed | SMR | Observed | SMR | Observed | SMR | Observed | SMR | Observed | SMR |
|  |  | (95% CI) |  | (95% CI) |  | (95% CI) |  | (95% CI) |  | (95% CI) |
| All causes of death | 70939 | **0.7(0.7-0.71)** | 4178 | **12.84(12.46-13.24)** | 21135 | **2.69(2.66-2.73)** | 26981 | **0.9(0.89-0.92)** | 18645 | **0.3(0.29-0.3)** |
| Non-cancer causes of death |  |  |  |  |  |  |  |  |  |  |
| Diseases of Heart | 21662 | **0.76(0.75-0.77)** | 1437 | **16.01(15.2-16.86)** | 6806 | **3.1(3.02-3.17)** | 8161 | **0.97(0.95-0.99)** | 5258 | **0.29(0.29-0.3)** |
| Chronic Obstructive Pulmonary Disease | 4519 | **0.69(0.67-0.71)** | 231 | **11.33(9.96-12.89)** | 1411 | **2.82(2.68-2.98)** | 1752 | **0.91(0.87-0.95)** | 1125 | **0.27(0.26-0.29)** |
| Cerebrovascular Diseases | 4199 | **0.79(0.76-0.81)** | 246 | **15.37(13.57-17.42)** | 1259 | **3.12(2.95-3.3)** | 1590 | 1.02(0.97-1.07) | 1104 | **0.33(0.31-0.35)** |
| Alzheimer’s | 2765 | **1.14(1.1-1.18)** | 60 | **9.67(7.51-12.45)** | 530 | **3.04(2.8-3.31)** | 1115 | **1.59(1.5-1.68)** | 1060 | **0.68(0.64-0.73)** |
| Diabetes Mellitus | 2334 | **0.74(0.71-0.77)** | 142 | **13.27(11.26-15.64)** | 760 | **3.05(2.84-3.27)** | 870 | 0.93(0.87-1) | 562 | **0.29(0.26-0.31)** |
| Pneumonia and Influenza | 1794 | **0.73(0.7-0.77)** | 80 | **11.43(9.18-14.23)** | 510 | **2.8(2.57-3.05)** | 731 | 1.02(0.95-1.1) | 473 | **0.31(0.28-0.34)** |
| Nephritis- Nephrotic Syndrome and Nephrosis | 1578 | **0.75(0.72-0.79)** | 77 | **12.16(9.73-15.2)** | 418 | **2.63(2.39-2.9)** | 640 | 1.04(0.96-1.13) | 443 | **0.34(0.31-0.37)** |
| Accidents and Adverse Effects | 2165 | **0.81(0.77-0.84)** | 150 | **15.79(13.46-18.53)** | 716 | **3.28(3.05-3.53)** | 799 | 1(0.93-1.07) | 500 | **0.3(0.28-0.33)** |
| Septicemia | 956 | **0.67(0.63-0.71)** | 70 | **15.27(12.08-19.3)** | 293 | **2.64(2.36-2.96)** | 362 | **0.86(0.77-0.95)** | 231 | **0.26(0.23-0.29)** |
| Hypertension without Heart Disease | 913 | 1(0.93-1.06) | 29 | **10.18(7.08-14.65)** | 268 | **3.81(3.38-4.29)** | 345 | **1.28(1.15-1.42)** | 271 | **0.47(0.42-0.53)** |
| Other Infectious Diseases | 526 | **1.95(1.79-2.12)** | 39 | **42.09(30.75-57.61)** | 172 | **8(6.89-9.29)** | 189 | **2.35(2.04-2.71)** | 126 | **0.75(0.63-0.9)** |
| In situ- benign or unknown behavior neoplasms | 253 | **0.36(0.32-0.41)** | 11 | **5.2(2.88-9.38)** | 75 | **1.42(1.14-1.79)** | 108 | **0.53(0.44-0.64)** | 59 | **0.14(0.1-0.17)** |
| Suicide and Self-Inflicted Injury | 703 | **0.87(0.81-0.94)** | 64 | **19.6(15.34-25.04)** | 251 | **3.61(3.19-4.09)** | 267 | 1.09(0.97-1.23) | 121 | **0.25(0.21-0.3)** |
| Chronic Liver Disease and Cirrhosis | 562 | **0.54(0.5-0.59)** | 34 | **7.61(5.44-10.65)** | 226 | **2.46(2.16-2.8)** | 176 | **0.55(0.48-0.64)** | 126 | **0.2(0.17-0.24)** |
| Aortic Aneurysm and Dissection | 421 | **0.65(0.59-0.71)** | 44 | **20.97(15.61-28.18)** | 157 | **3.12(2.67-3.65)** | 148 | **0.77(0.66-0.91)** | 72 | **0.18(0.14-0.22)** |
| Other Diseases of Arteries- Arterioles- Capillaries | 286 | **0.72(0.64-0.8)** | 18 | **14.54(9.16-23.08)** | 90 | **2.95(2.4-3.63)** | 107 | **0.91(0.75-1.1)** | 71 | **0.28(0.22-0.36)** |
| Atherosclerosis | 258 | **0.73(0.64-0.82)** | 15 | **15.04(9.07-24.95)** | 89 | **3.39(2.75-4.17)** | 86 | **0.83(0.67-1.03)** | 68 | **0.3(0.24-0.38)** |
| Other cancers (Non-prostate) causes of death | 2445 | **0.1(0.1-0.11)** | 118 | **1.44(1.2-1.73)** | 695 | **0.37(0.34-0.4)** | 942 | **0.14(0.13-0.14)** | 690 | **0.05(0.04-0.05)** |

Bolded SMRs are significantly different from 1.00 (P < .05). A blank indicates that because of 0 observed deaths, the SMR, 95% CI, and P value were not calculated.

**Supplementary Table 17.**Standardized-mortality ratios (SMRs) for each cause of death following prostate cancer diagnosis in males with grade III

| Cause of death | **Total** | | **< 1 Year** | | **1-5 Years** | | **5-10 Years** | | **≥10 Years** | |
| --- | --- | --- | --- | --- | --- | --- | --- | --- | --- | --- |
|  | Observed | **SMR** | Observed | **SMR** | Observed | **SMR** | Observed | **SMR** | Observed | **SMR** |
|  |  | **(95% CI)** |  | **(95% CI)** |  | **(95% CI)** |  | **(95% CI)** |  | **(95% CI)** |
| All causes of death | 78976 | **1.11(1.11-1)** | 8993 | **19.56(19.16-19.97)** | 36936 | **3.26(3.22-3.29)** | 25323 | **0.78(0.77-0.79)** | 7724 | **0.29(0.28-0.3)** |
| Non-cancer causes of death |  |  |  |  |  |  |  |  |  |  |
| Diseases of Heart | 16711 | **0.83(0.82-0.84)** | 1753 | **13.11(12.51-13.74)** | 7253 | **2.24(2.18-2.29)** | 5764 | **0.62(0.61-0.64)** | 1941 | **0.26(0.25-0.27)** |
| Chronic Obstructive Pulmonary Disease | 3475 | **0.76(0.74-0.79)** | 288 | **9.89(8.81-11.1)** | 1512 | **2.1(1.99-2.2)** | 1287 | **0.61(0.58-0.65)** | 388 | **0.23(0.2-0.25)** |
| Cerebrovascular Diseases | 3095 | **0.82(0.79-0.85)** | 293 | **11.48(10.24-12.87)** | 1296 | **2.13(2.01-2.25)** | 1149 | **0.67(0.63-0.71)** | 357 | **0.25(0.23-0.28)** |
| Alzheimer’s | 1766 | **1(0.95-1.04)** | 52 | **3.9(2.97-5.12)** | 596 | **2.02(1.86-2.19)** | 801 | 1(0.94-1.08) | 317 | **0.48(0.43-0.53)** |
| Diabetes Mellitus | 1812 | **0.83(0.79-0.87)** | 166 | **12.37(10.63-14.4)** | 766 | **2.23(2.08-2.39)** | 680 | **0.67(0.63-0.73)** | 200 | **0.25(0.21-0.28)** |
| Pneumonia and Influenza | 1411 | **0.8(0.76-0.85)** | 123 | **9.88(8.28-11.78)** | 574 | **1.99(1.84-2.16)** | 526 | **0.66(0.61-0.72)** | 188 | **0.29(0.25-0.33)** |
| Nephritis- Nephrotic Syndrome and Nephrosis | 1134 | **0.77(0.72-0.81)** | 96 | **9.63(7.88-11.76)** | 454 | **1.9(1.73-2.08)** | 439 | **0.65(0.59-0.71)** | 145 | **0.26(0.22-0.31)** |
| Accidents and Adverse Effects | 1716 | **0.91(0.87-0.96)** | 169 | **14.04(12.08-16.33)** | 790 | **2.61(2.43-2.8)** | 571 | **0.66(0.61-0.71)** | 186 | **0.27(0.23-0.31)** |
| Septicemia | 856 | **0.85(0.8-0.91)** | 86 | **13.28(10.75-16.41)** | 382 | **2.39(2.16-2.64)** | 301 | **0.65(0.58-0.73)** | 87 | **0.23(0.19-0.29)** |
| Hypertension without Heart Disease | 739 | **1.13(1.06-1.22)** | 78 | **17.69(14.17-22.09)** | 295 | **2.79(2.49-3.13)** | 283 | 0.95(0.85-1.07) | 83 | **0.34(0.28-0.42)** |
| Other Infectious Diseases | 438 | **2.34(2.13-2.57)** | 42 | **36.36(26.87-49.2)** | 212 | **7.17(6.26-8.2)** | 146 | **1.69(1.43-1.98)** | 38 | **0.54(0.4-0.75)** |
| In situ- benign or unknown behavior neoplasms | 187 | **0.38(0.33-0.44)** | 22 | **6.87(4.52-10.43)** | 65 | 0.84(0.66-1.07) | 69 | **0.31(0.24-0.39)** | 31 | **0.17(0.12-0.24)** |
| Suicide and Self-Inflicted Injury | 583 | 1.06(0.98-1.15) | 92 | **29.1(23.72-35.7)** | 253 | **2.91(2.58-3.3)** | 194 | **0.75(0.65-0.87)** | 44 | **0.22(0.16-0.29)** |
| Chronic Liver Disease and Cirrhosis | 466 | **0.66(0.61-0.73)** | 36 | **9.38(6.76-13)** | 251 | **2.28(2.01-2.58)** | 139 | **0.42(0.35-0.49)** | 40 | **0.16(0.11-0.21)** |
| Aortic Aneurysm and Dissection | 308 | **0.68(0.61-0.76)** | 39 | **13.74(10.04-18.8)** | 148 | **2.08(1.77-2.44)** | 96 | **0.46(0.38-0.56)** | 25 | **0.15(0.1-0.22)** |
| Other Diseases of Arteries- Arterioles- Capillaries | 201 | **0.72(0.62-0.82)** | 14 | **7.58(4.49-12.8)** | 94 | **2.09(1.71-2.56)** | 64 | **0.5(0.39-0.64)** | 29 | **0.28(0.19-0.4)** |
| Atherosclerosis | 208 | **0.81(0.71-0.93)** | 13 | **7.1(4.12-12.23)** | 73 | **1.74(1.38-2.18)** | 92 | **0.79(0.65-0.97)** | 30 | **0.31(0.22-0.45)** |
| Other cancers (Non-prostate) causes of death | 2239 | **0.14(0.13-0.15)** | 233 | **2.44(2.15-2.77)** | 954 | **0.38(0.36-0.41)** | 788 | **0.11(0.1-0.11)** | 264 | **0.04(0.04-0.05)** |

Bolded SMRs are significantly different from 1.00 (P < .05). A blank indicates that because of 0 observed deaths, the SMR, 95% CI, and P value were not calculated.

**Supplementary Table 18.** Standardized-mortality ratios (SMRs) for each cause of death following prostate cancer diagnosis in males with grade Ⅳ

| Cause of death | **Total** | | **< 1 Year** | | **1-5 Years** | | **5-10 Years** | | **≥10 Years** | |
| --- | --- | --- | --- | --- | --- | --- | --- | --- | --- | --- |
|  | Observed | SMR | Observed | SMR | Observed | SMR | Observed | SMR | Observed | SMR |
|  |  | (95% CI) |  | (95% CI) |  | (95% CI) |  | (95% CI) |  | (95% CI) |
| All causes of death | 965 | **2.26(2.12-2.41)** | 214 | **31.55(27.6-36.08)** | 476 | **5.44(4.98-5.96)** | 205 | **1.2(1.04-1.37)** | 70 | **0.43(0.34-0.55)** |
| Non-cancer causes of death |  |  |  |  |  |  |  |  |  |  |
| Diseases of Heart | 136 | **1.11(0.93-1.31)** | 17 | **8.34(5.19-13.42)** | 60 | **2.33(1.81-3)** | 42 | 0.85(0.63-1.15) | 17 | **0.37(0.23-0.6)** |
| Chronic Obstructive Pulmonary Disease | 28 | 1.02(0.7-1.47) | 1 | — | 12 | **2.17(1.23-3.82)** | 12 | 1.08(0.62-1.91) | 3 | — |
| Cerebrovascular Diseases | 25 | 1.06(0.72-1.57) | 5 | — | 12 | **2.41(1.37-4.24)** | 7 | — | 1 | — |
| Alzheimer’s | 14 | 1.18(0.7-1.99) | 2 | — | 4 | — | 4 | — | 4 | — |
| Diabetes Mellitus | 14 | 1.1(0.65-1.86) | 3 | — | 4 | — | 5 | — | 2 | — |
| Pneumonia and Influenza | 7 | — | 0 | — | 3 | — | 3 | — | 1 | — |
| Nephritis- Nephrotic Syndrome and Nephrosis | 9 | — | 0 | — | 5 | — | 2 | — | 2 | — |
| Accidents and Adverse Effects | 11 | 1(0.55-1.8) | 2 | — | 7 | — | 1 | — | 1 | — |
| Septicemia | 7 | — | 0 | — | 5 | — | 1 | — | 1 | — |
| Hypertension without Heart Disease | 7 | — | 1 | — | 1 | — | 4 | — | 1 | — |
| Other Infectious Diseases | 4 | — | 0 | — | 1 | — | 2 | — | 1 | — |
| In situ- benign or unknown behavior neoplasms | 1 | — | 0 | — | 1 | — | 0 | — | 0 | — |
| Suicide and Self-Inflicted Injury | 7 | — | 2 | — | 4 | — | 1 | — | 0 | — |
| Chronic Liver Disease and Cirrhosis | 2 | — | 0 | — | 2 | — | 0 | — | 0 | — |
| Aortic Aneurysm and Dissection | 0 | — | 0 | — | 0 | — | 0 | — | 0 | — |
| Other Diseases of Arteries- Arterioles- Capillaries | 5 | — | 1 | — | 2 | — | 2 | — | 0 | — |
| Atherosclerosis | 2 | — | 1 | — | 0 | — | 1 | — | 0 | — |
| Other cancers (Non-prostate) causes of death | 47 | **0.51(0.39-0.68)** | 22 | **17.89(11.78-27.17)** | 18 | 1.04(0.66-1.66) | 6 | — | 1 | — |

Bolded SMRs are significantly different from 1.00 (P < .05). A blank indicates that because of 0 observed deaths, the SMR, 95% CI, and P value were not calculated.

**Supplementary Table 19.**  Standardized-mortality ratios (SMRs) for each cause of death following prostate cancer diagnosis in males with year of diagnosis between 2000-2005

| Cause of death | **Total** | | **< 1 Year** | | **1-5 Years** | | **5-10 Years** | | **≥10 Years** | |
| --- | --- | --- | --- | --- | --- | --- | --- | --- | --- | --- |
|  | Observed | SMR | Observed | SMR | Observed | SMR | Observed | SMR | Observed | SMR |
|  |  | (95% CI) |  | (95% CI) |  | (95% CI) |  | (95% CI) |  | (95% CI) |
| All causes of death | 106394 | **0.97(0.97,0.98)** | 8591 | **30.54(29.9,31.19)** | 32834 | **5.52(5.46,5.58)** | 36823 | **2.09(2.07,2.11)** | 28146 | **0.33(0.33,0.33)** |
| Non-cancer causes of death |  |  |  |  |  |  |  |  |  |  |
| Diseases of Heart | 27902 | **0.89(0.88,0.9)** | 2054 | **24.35(23.32,25.43)** | 8258 | **4.67(4.57,4.77)** | 9964 | **1.91(1.87,1.95)** | 7626 | **0.31(0.31,0.32)** |
| Chronic Obstructive Pulmonary Disease | 5795 | **0.82(0.8,0.84)** | 334 | **18.85(16.94,20.99)** | 1702 | **4.48(4.27,4.7)** | 2158 | **1.9(1.82,1.98)** | 1601 | **0.29(0.27,0.3)** |
| Cerebrovascular Diseases | 5367 | **0.9(0.87,0.92)** | 349 | **21.03(18.94,23.36)** | 1580 | **4.54(4.32,4.77)** | 1882 | **1.83(1.75,1.92)** | 1556 | **0.34(0.32,0.36)** |
| Alzheimer’s | 3472 | **1.17(1.14,1.21)** | 76 | **7.77(6.21,9.73)** | 617 | **3.09(2.86,3.34)** | 1284 | **2.21(2.1,2.34)** | 1495 | **0.69(0.66,0.73)** |
| Diabetes Mellitus | 2888 | **0.88(0.85,0.91)** | 161 | **21.2(18.16,24.74)** | 861 | **5.26(4.92,5.62)** | 1061 | **2.16(2.04,2.3)** | 805 | **0.31(0.29,0.33)** |
| Pneumonia and Influenza | 2494 | **0.88(0.85,0.92)** | 165 | **19.32(16.59,22.51)** | 680 | **3.86(3.58,4.16)** | 942 | **1.83(1.72,1.95)** | 707 | **0.33(0.31,0.36)** |
| Nephritis, Nephrotic Syndrome and Nephrosis | 1992 | **0.85(0.82,0.89)** | 104 | **16.13(13.31,19.55)** | 513 | **3.79(3.48,4.14)** | 744 | **1.87(1.74,2)** | 631 | **0.35(0.33,0.38)** |
| Accidents and Adverse Effects | 2444 | **0.87(0.83,0.9)** | 131 | **18.57(15.65,22.04)** | 706 | **4.76(4.42,5.13)** | 891 | **2.04(1.91,2.18)** | 716 | **0.32(0.3,0.35)** |
| Septicemia | 1279 | **0.83(0.78,0.87)** | 95 | **24.05(19.67,29.4)** | 376 | **4.48(4.05,4.96)** | 467 | **1.87(1.71,2.05)** | 341 | **0.28(0.25,0.31)** |
| Hypertension without Heart Disease | 1130 | **1.11(1.05,1.18)** | 45 | **15.83(11.82,21.2)** | 266 | **4.49(3.98,5.07)** | 436 | **2.51(2.28,2.76)** | 383 | **0.49(0.44,0.54)** |
| Other Infectious Diseases | 618 | **2.2(2.03,2.38)** | 40 | **61.16(44.86,83.38)** | 178 | **12.65(10.92,14.65)** | 228 | **5.42(4.76,6.18)** | 172 | **0.77(0.66,0.89)** |
| In situ, benign or unknown behavior neoplasms | 345 | **0.45(0.41,0.5)** | 17 | **8.42(5.23,13.54)** | 94 | **2.2(1.79,2.69)** | 139 | 1.09(0.93,1.29) | 95 | **0.16(0.13,0.2)** |
| Suicide and Self-Inflicted Injury | 753 | 0.98(0.91,1.05) | 67 | **44.61(35.11,56.67)** | 215 | **6.57(5.75,7.51)** | 298 | **3.05(2.73,3.42)** | 173 | **0.27(0.23,0.31)** |
| Chronic Liver Disease and Cirrhosis | 645 | **0.68(0.63,0.73)** | 40 | **25.28(18.54,34.46)** | 234 | **6.64(5.84,7.55)** | 197 | **1.86(1.62,2.14)** | 174 | **0.21(0.19,0.25)** |
| Aortic Aneurysm and Dissection | 503 | **0.73(0.67,0.79)** | 52 | **30.97(23.6,40.64)** | 182 | **5.04(4.36,5.83)** | 168 | **1.55(1.34,1.81)** | 101 | **0.18(0.15,0.22)** |
| Other Diseases of Arteries, Arterioles, Capillaries | 367 | **0.84(0.76,0.93)** | 24 | **20.69(13.87,30.87)** | 101 | **4.13(3.39,5.01)** | 133 | **1.83(1.55,2.17)** | 109 | **0.32(0.27,0.39)** |
| Atherosclerosis | 370 | **0.89(0.81,0.99)** | 25 | **19.68(13.3,29.13)** | 121 | **4.63(3.88,5.53)** | 124 | **1.63(1.36,1.94)** | 100 | **0.32(0.27,0.39)** |
| Other cancers (Non-prostate) causes of death | 3581 | **0.15(0.15,0.16)** | 265 | **5.19(4.6,5.85)** | 1065 | 0.95(0.9,1.01) | 1232 | **0.37(0.35,0.39)** | 1019 | **0.05(0.05,0.06)** |

Bolded SMRs are significantly different from 1.00 (P < .05). A blank indicates that because of 0 observed deaths, the SMR, 95% CI, and P value were not calculated.

**Supplementary Table 20.** Standardized-mortality ratios (SMRs) for each cause of death following prostate cancer diagnosis in males with year of diagnosis between 2006-2016

| Cause of death | **Total** | | **< 1 Year** | | **1-5 Years** | | **5-10 Years** | | **≥10 Years** | |
| --- | --- | --- | --- | --- | --- | --- | --- | --- | --- | --- |
|  | Observed | SMR | Observed | SMR | Observed | SMR | Observed | SMR | Observed | SMR |
|  |  | (95% CI) |  | (95% CI) |  | (95% CI) |  | (95% CI) |  | (95% CI) |
| All causes of death | 70728 | **0.91(0.9,0.92)** | 12673 | **13.09(12.87,13.32)** | 37167 | **2.13(2.11,2.16)** | 20327 | **0.41(0.41,0.42)** | 561 | **0.05(0.05,0.06)** |
| Non-cancer causes of death |  |  |  |  |  |  |  |  |  |  |
| Diseases of Heart | 15194 | **0.7(0.69,0.71)** | 2116 | **7.64(7.32,7.97)** | 7758 | **1.59(1.55,1.62)** | 5149 | **0.38(0.37,0.39)** | 171 | **0.06(0.05,0.07)** |
| Chronic Obstructive Pulmonary Disease | 3140 | **0.63(0.61,0.65)** | 363 | **6.05(5.46,6.71)** | 1617 | **1.47(1.4,1.54)** | 1118 | **0.35(0.33,0.38)** | 42 | **0.06(0.05,0.09)** |
| Cerebrovascular Diseases | 2839 | **0.71(0.69,0.74)** | 382 | **7.48(6.77,8.27)** | 1346 | **1.5(1.43,1.59)** | 1089 | **0.43(0.41,0.46)** | 22 | **0.04(0.03,0.06)** |
| Alzheimer’s | 1658 | **0.98(0.94,1.03)** | 136 | **5.57(4.71,6.58)** | 706 | **1.81(1.68,1.94)** | 795 | **0.75(0.7,0.81)** | 21 | **0.1(0.06,0.15)** |
| Diabetes Mellitus | 1717 | **0.69(0.66,0.72)** | 239 | **8.09(7.12,9.18)** | 861 | **1.56(1.46,1.67)** | 602 | **0.38(0.35,0.41)** | 15 | **0.05(0.03,0.08)** |
| Pneumonia and Influenza | 1143 | **0.64(0.6,0.68)** | 138 | **5.69(4.81,6.72)** | 566 | **1.39(1.28,1.51)** | 429 | **0.38(0.35,0.42)** | 10 | **0.04(0.02,0.08)** |
| Nephritis, Nephrotic Syndrome and Nephrosis | 1054 | **0.67(0.63,0.71)** | 123 | **6.12(5.13,7.3)** | 509 | **1.44(1.32,1.58)** | 409 | **0.41(0.38,0.46)** | 13 | **0.06(0.04,0.11)** |
| Accidents and Adverse Effects | 1814 | **0.85(0.81,0.89)** | 260 | **9.61(8.51,10.86)** | 973 | **2(1.88,2.13)** | 563 | **0.42(0.39,0.45)** | 18 | **0.06(0.04,0.1)** |
| Septicemia | 801 | **0.73(0.68,0.78)** | 111 | **8.18(6.79,9.85)** | 432 | **1.76(1.6,1.93)** | 250 | **0.36(0.32,0.41)** | 8 | — |
| Hypertension without Heart Disease | 723 | 1.04(0.97,1.12) | 91 | **10.06(8.19,12.35)** | 377 | **2.4(2.17,2.65)** | 250 | **0.57(0.51,0.65)** | 5 | — |
| Other Infectious Diseases | 466 | **2.18(1.99,2.39)** | 73 | **28.53(22.69,35.89)** | 259 | **5.45(4.82,6.15)** | 131 | **0.97(0.82,1.15)** | 3 | — |
| In situ, benign or unknown behavior neoplasms | 155 | **0.3(0.25,0.35)** | 25 | **3.85(2.6,5.7)** | 73 | **0.63(0.5,0.79)** | 55 | **0.17(0.13,0.22)** | 2 | — |
| Suicide and Self-Inflicted Injury | 652 | 0.95(0.88,1.03) | 118 | **14.6(12.19,17.49)** | 345 | **2.24(2.02,2.49)** | 186 | **0.43(0.37,0.5)** | 3 | — |
| Chronic Liver Disease and Cirrhosis | 471 | **0.52(0.47,0.57)** | 49 | **4.66(3.52,6.17)** | 286 | **1.4(1.25,1.57)** | 134 | **0.23(0.2,0.28)** | 2 | — |
| Aortic Aneurysm and Dissection | 285 | **0.57(0.51,0.64)** | 43 | **7.17(5.31,9.66)** | 150 | **1.35(1.15,1.59)** | 90 | **0.28(0.23,0.35)** | 2 | — |
| Other Diseases of Arteries, Arterioles, Capillaries | 183 | **0.61(0.52,0.7)** | 19 | **5.01(3.2,7.85)** | 115 | **1.7(1.42,2.04)** | 48 | **0.25(0.19,0.33)** | 1 | — |
| Atherosclerosis | 179 | **0.7(0.6,0.81)** | 26 | **7.35(5.01,10.8)** | 75 | **1.27(1.02,1.6)** | 74 | **0.46(0.37,0.58)** | 4 | — |
| Other cancers (Non-prostate) causes of death | 2043 | **0.11(0.1,0.11)** | 427 | **1.97(1.8,2.17)** | 947 | **0.23(0.22,0.24)** | 647 | **0.05(0.05,0.06)** | 22 | **0.01(0.01,0.01)** |

Bolded SMRs are significantly different from 1.00 (P < .05). A blank indicates that because of 0 observed deaths, the SMR, 95% CI, and P value were not calculated.

**Supplementary Table 21** . Standardized-mortality ratios (SMRs) for each cause of death following prostate cancer diagnosis in males with non treatment

| Cause of death | **Total** | | **< 1 Year** | | **1-5 Years** | | **5-10 Years** | | **≥10 Years** | |
| --- | --- | --- | --- | --- | --- | --- | --- | --- | --- | --- |
|  | Observed | SMR | Observed | SMR | Observed | SMR | Observed | SMR | Observed | SMR |
|  |  | (95% CI) |  | (95% CI) |  | (95% CI) |  | (95% CI) |  | (95% CI) |
| All causes of death | 95481 | **1.36(1.35-1.37)** | 15403 | **19.21(18.91-19.51)** | 43507 | **3.2(3.17-3.23)** | 26786 | **0.93(0.92-0.95)** | 9785 | **0.36(0.36-0.37)** |
| Non-cancer causes of death |  |  |  |  |  |  |  |  |  |  |
| Diseases of Heart | 22245 | **1.08(1.06-1.09)** | 3043 | **12.73(12.29-13.19)** | 9443 | **2.36(2.31-2.4)** | 7074 | **0.84(0.82-0.86)** | 2685 | **0.34(0.33-0.35)** |
| Chronic Obstructive Pulmonary Disease | 4657 | **1.04(1.01-1.07)** | 557 | **11.19(10.3-12.16)** | 2094 | **2.44(2.34-2.55)** | 1490 | **0.82(0.77-0.86)** | 516 | **0.3(0.27-0.33)** |
| Cerebrovascular Diseases | 4280 | **1.07(1.04-1.1)** | 553 | **11.97(11.01-13.01)** | 1822 | **2.36(2.25-2.47)** | 1395 | **0.85(0.81-0.9)** | 510 | **0.33(0.3-0.36)** |
| Alzheimer’s | 2816 | **1.27(1.23-1.32)** | 198 | **7.47(6.5-8.59)** | 958 | **2.25(2.11-2.4)** | 1118 | **1.23(1.16-1.31)** | 542 | **0.63(0.58-0.69)** |
| Diabetes Mellitus | 2223 | **1.12(1.08-1.17)** | 288 | **13.01(11.59-14.6)** | 940 | **2.44(2.29-2.6)** | 734 | **0.91(0.84-0.97)** | 261 | **0.34(0.3-0.39)** |
| Pneumonia and Influenza | 2084 | **1.04(1-1.09)** | 253 | **10.7(9.46-12.11)** | 847 | **2.19(2.05-2.34)** | 721 | **0.88(0.82-0.95)** | 263 | **0.34(0.3-0.38)** |
| Nephritis- Nephrotic Syndrome and Nephrosis | 1574 | 1.01(0.96-1.06) | 193 | **10.7(9.3-12.33)** | 637 | **2.11(1.95-2.28)** | 536 | **0.84(0.77-0.91)** | 208 | **0.35(0.3-0.4)** |
| Accidents and Adverse Effects | 1902 | **1.07(1.02-1.12)** | 260 | **12.53(11.09-14.15)** | 812 | **2.31(2.16-2.48)** | 592 | **0.82(0.75-0.88)** | 238 | **0.35(0.31-0.4)** |
| Septicemia | 1057 | **1.07(1.01-1.14)** | 144 | **12.83(10.9-15.11)** | 488 | **2.55(2.33-2.79)** | 312 | **0.77(0.69-0.86)** | 113 | **0.3(0.25-0.36)** |
| Hypertension without Heart Disease | 973 | **1.42(1.33-1.51)** | 107 | **13.31(11.01-16.09)** | 381 | **2.85(2.58-3.16)** | 345 | **1.23(1.11-1.37)** | 140 | **0.53(0.45-0.63)** |
| Other Infectious Diseases | 502 | **2.95(2.71-3.22)** | 80 | **41.87(33.63-52.12)** | 230 | **6.93(6.09-7.89)** | 139 | **2(1.69-2.36)** | 53 | **0.81(0.62-1.06)** |
| In situ- benign or unknown behavior neoplasms | 251 | **0.5(0.45-0.57)** | 36 | **6.38(4.61-8.85)** | 94 | **0.98(0.8-1.2)** | 91 | **0.45(0.36-0.55)** | 30 | **0.16(0.11-0.22)** |
| Suicide and Self-Inflicted Injury | 552 | **1.31(1.2-1.42)** | 97 | **20.14(16.5-24.57)** | 250 | **2.92(2.58-3.31)** | 161 | **0.93(0.8-1.09)** | 44 | **0.27(0.2-0.37)** |
| Chronic Liver Disease and Cirrhosis | 415 | **0.86(0.78-0.94)** | 68 | **12.4(9.78-15.73)** | 215 | **2.15(1.88-2.46)** | 95 | **0.48(0.39-0.59)** | 37 | **0.2(0.15-0.28)** |
| Aortic Aneurysm and Dissection | 372 | **0.87(0.78-0.96)** | 71 | **14.86(11.78-18.75)** | 184 | **2.22(1.92-2.57)** | 98 | **0.56(0.46-0.68)** | 19 | **0.11(0.07-0.18)** |
| Other Diseases of Arteries- Arterioles- Capillaries | 282 | 0.99(0.88-1.11) | 34 | **10.4(7.43-14.56)** | 130 | **2.35(1.98-2.8)** | 85 | **0.73(0.59-0.9)** | 33 | **0.3(0.21-0.42)** |
| Atherosclerosis | 332 | **1.12(1.01-1.25)** | 41 | **11.69(8.61-15.88)** | 142 | **2.49(2.11-2.93)** | 107 | **0.89(0.73-1.07)** | 42 | **0.37(0.27-0.5)** |
| Other cancers (Non-prostate) causes of death | 2640 | **0.19(0.18-0.2)** | 480 | **3.17(2.9-3.47)** | 1119 | **0.42(0.39-0.44)** | 730 | **0.13(0.12-0.14)** | 311 | **0.06(0.05-0.07)** |

Bolded SMRs are significantly different from 1.00 (P < .05). A blank indicates that because of 0 observed deaths, the SMR, 95% CI, and P value were not calculated.

**Supplementary Table 22.**  Standardized-mortality ratios (SMRs) for each cause of death following prostate cancer diagnosis in males with radical prostatectomy (RP) ± radiotherapy/ chemotherapy

| Cause of death | **Total** | | **< 1 Year** | | **1-5 Years** | | **5-10 Years** | | **≥10 Years** | |
| --- | --- | --- | --- | --- | --- | --- | --- | --- | --- | --- |
|  | Observed | SMR | Observed | SMR | Observed | SMR | Observed | SMR | Observed | SMR |
|  |  | (95% CI) |  | (95% CI) |  | (95% CI) |  | (95% CI) |  | (95% CI) |
| All causes of death | 18532 | **0.44(0.44-0.45)** | 889 | **7.09(6.64-7.57)** | 4904 | **1.63(1.59-1.68)** | 7293 | **0.58(0.57-0.59)** | 5446 | **0.21(0.2-0.22)** |
| Non-cancer causes of death |  |  |  |  |  |  |  |  |  |  |
| Diseases of Heart | 4529 | **0.41(0.4-0.42)** | 306 | **9.36(8.37-10.47)** | 1252 | **1.59(1.51-1.69)** | 1674 | **0.51(0.48-0.53)** | 1297 | **0.19(0.18-0.2)** |
| Chronic Obstructive Pulmonary Disease | 817 | **0.31(0.29-0.33)** | 10 | 1.42(0.77-2.64) | 140 | **0.8(0.68-0.95)** | 349 | **0.46(0.41-0.51)** | 318 | **0.19(0.17-0.21)** |
| Cerebrovascular Diseases | 928 | **0.49(0.46-0.53)** | 48 | **9.63(7.26-12.78)** | 217 | **1.76(1.54-2.01)** | 343 | **0.64(0.57-0.71)** | 320 | **0.26(0.24-0.29)** |
| Alzheimer’s | 482 | **0.91(0.84-1)** | 0 | — | 46 | **1.93(1.45-2.58)** | 181 | **1.49(1.29-1.72)** | 255 | **0.67(0.59-0.76)** |
| Diabetes Mellitus | 522 | **0.35(0.33-0.39)** | 25 | **5.36(3.62-7.93)** | 125 | **1.13(0.95-1.35)** | 202 | **0.44(0.38-0.51)** | 170 | **0.19(0.16-0.22)** |
| Pneumonia and Influenza | 292 | **0.39(0.35-0.44)** | 5 | — | 55 | **1.18(0.91-1.54)** | 117 | **0.57(0.47-0.68)** | 115 | **0.23(0.19-0.28)** |
| Nephritis- Nephrotic Syndrome and Nephrosis | 286 | **0.38(0.34-0.43)** | 2 | — | 59 | **1.18(0.91-1.52)** | 103 | **0.47(0.39-0.57)** | 122 | **0.25(0.21-0.3)** |
| Accidents and Adverse Effects | 771 | **0.6(0.56-0.65)** | 36 | **7.73(5.58-10.72)** | 292 | **2.76(2.46-3.1)** | 267 | **0.65(0.58-0.73)** | 176 | **0.23(0.2-0.27)** |
| Septicemia | 207 | **0.35(0.31-0.4)** | 12 | **6.95(3.95-12.24)** | 45 | 1.08(0.81-1.45) | 96 | **0.54(0.45-0.66)** | 54 | **0.15(0.11-0.19)** |
| Hypertension without Heart Disease | 209 | **0.61(0.53-0.7)** | 7 | — | 57 | **2.37(1.82-3.07)** | 78 | **0.77(0.62-0.96)** | 67 | **0.31(0.24-0.39)** |
| Other Infectious Diseases | 157 | **1.23(1.05-1.44)** | 9 | — | 56 | **5.76(4.43-7.48)** | 57 | **1.43(1.1-1.85)** | 35 | **0.45(0.32-0.63)** |
| In situ- benign or unknown behavior neoplasms | 47 | **0.18(0.14-0.24)** | 0 | — | 12 | 0.7(0.4-1.23) | 21 | **0.28(0.18-0.43)** | 14 | **0.08(0.05-0.14)** |
| Suicide and Self-Inflicted Injury | 378 | **0.76(0.69-0.84)** | 38 | **19.1(13.9-26.24)** | 143 | **3.22(2.74-3.8)** | 144 | 0.86(0.73-1.01) | 53 | **0.19(0.14-0.25)** |
| Chronic Liver Disease and Cirrhosis | 253 | **0.36(0.32-0.4)** | — | **1.72(0.72-4.13)** | 96 | **1.49(1.22-1.82)** | 89 | **0.37(0.3-0.45)** | 63 | **0.16(0.12-0.2)** |
| Aortic Aneurysm and Dissection | 119 | **0.43(0.36-0.51)** | — | **3.74(1.21-11.59)** | 41 | **2.1(1.55-2.86)** | 41 | **0.49(0.36-0.67)** | 34 | **0.2(0.14-0.27)** |
| Other Diseases of Arteries- Arterioles- Capillaries | 67 | **0.44(0.34-0.55)** | — | **7.03(2.27-21.8)** | 17 | **1.63(1.01-2.62)** | 22 | **0.49(0.32-0.74)** | 25 | **0.26(0.17-0.38)** |
| Atherosclerosis | 48 | **0.46(0.35-0.62)** | — | 4.08(0.58-28.99) | 11 | **1.77(0.98-3.2)** | 22 | 0.79(0.52-1.19) | 14 | **0.2(0.12-0.34)** |
| Other cancers (Non-prostate) causes of death | 833 | **0.07(0.07-0.08)** | 24 | **0.64(0.43-0.95)** | 208 | **0.23(0.2-0.27)** | 328 | **0.09(0.08-0.1)** | 273 | **0.04(0.03-0.04)** |

Bolded SMRs are significantly different from 1.00 (P < .05). A blank indicates that because of 0 observed deaths, the SMR, 95% CI, and P value were not calculated

**Supplementary Table 23.**  Standardized-mortality ratios (SMRs) for each cause of death following prostate cancer diagnosis in males with radiation ± /chemotherapy

| Cause of death | **Total** | | **< 1 Year** | | **1-5 Years** | | **5-10 Years** | | **≥10 Years** | |
| --- | --- | --- | --- | --- | --- | --- | --- | --- | --- | --- |
|  | Observed | SMR | Observed | SMR | Observed | SMR | Observed | SMR | Observed | SMR |
|  |  | (95% CI) |  | (95% CI) |  | (95% CI) |  | (95% CI) |  | (95% CI) |
| All causes of death | 59788 | **0.82(0.81-0.83)** | 4264 | **14.93(14.49-15.38)** | 20189 | **3.22(3.18-3.26)** | 22213 | **0.91(0.9-0.92)** | 13122 | **0.31(0.31-0.32)** |
| Non-cancer causes of death |  |  |  |  |  |  |  |  |  |  |
| Diseases of Heart | 15645 | **0.76(0.75-0.77)** | 739 | **9.39(8.74-10.1)** | 5038 | **2.92(2.84-3)** | 6143 | **0.9(0.87-0.92)** | 3725 | **0.31(0.3-0.32)** |
| Chronic Obstructive Pulmonary Disease | 3279 | **0.68(0.66-0.7)** | 105 | **5.63(4.65-6.82)** | 1010 | **2.42(2.28-2.58)** | 1388 | **0.85(0.8-0.89)** | 776 | **0.28(0.26-0.3)** |
| Cerebrovascular Diseases | 2870 | **0.73(0.7-0.76)** | 113 | **7.87(6.54-9.46)** | 835 | **2.62(2.45-2.8)** | 1193 | **0.92(0.87-0.97)** | 729 | **0.32(0.3-0.34)** |
| Alzheimer’s | 1755 | **0.96(0.91-1)** | 6 | — | 291 | **2.3(2.05-2.58)** | 751 | **1.3(1.21-1.4)** | 707 | **0.63(0.58-0.68)** |
| Diabetes Mellitus | 1788 | **0.8(0.76-0.84)** | 81 | **8.65(6.96-10.75)** | 619 | **3.02(2.79-3.27)** | 706 | **0.92(0.85-0.99)** | 382 | **0.3(0.27-0.34)** |
| Pneumonia and Influenza | 1197 | **0.66(0.63-0.7)** | 34 | **5.47(3.91-7.66)** | 319 | **2.32(2.08-2.59)** | 510 | **0.88(0.8-0.96)** | 334 | **0.31(0.28-0.35)** |
| Nephritis- Nephrotic Syndrome and Nephrosis | 1126 | **0.73(0.69-0.78)** | 28 | **4.95(3.42-7.17)** | 300 | **2.39(2.14-2.68)** | 494 | **0.97(0.89-1.06)** | 304 | **0.34(0.3-0.38)** |
| Accidents and Adverse Effects | 1515 | **0.82(0.78-0.87)** | 84 | **10.84(8.75-13.42)** | 549 | **3.33(3.06-3.62)** | 573 | 0.92(0.85-1) | 309 | **0.3(0.26-0.33)** |
| Septicemia | 782 | **0.75(0.7-0.81)** | 47 | **11.56(8.69-15.39)** | 256 | **2.86(2.53-3.23)** | 300 | **0.86(0.76-0.96)** | 179 | **0.3(0.26-0.35)** |
| Hypertension without Heart Disease | 646 | **0.98(0.91-1.06)** | 21 | **8.47(5.52-12.99)** | 190 | **3.5(3.04-4.04)** | 256 | **1.17(1.04-1.33)** | 179 | **0.46(0.4-0.54)** |
| Other Infectious Diseases | 409 | **2.14(1.94-2.36)** | 24 | **29.89(20.04-44.6)** | 144 | **8.22(6.99-9.68)** | 157 | **2.39(2.05-2.8)** | 84 | **0.78(0.63-0.97)** |
| In situ- benign or unknown behavior neoplasms | 196 | **0.38(0.33-0.44)** | 6 | — | 58 | **1.36(1.05-1.76)** | 81 | **0.47(0.38-0.59)** | 51 | **0.17(0.13-0.23)** |
| Suicide and Self-Inflicted Injury | 451 | **0.87(0.79-0.95)** | 46 | **17.92(13.42-23.92)** | 154 | **2.88(2.46-3.37)** | 174 | 0.95(0.82-1.1) | 77 | **0.27(0.22-0.34)** |
| Chronic Liver Disease and Cirrhosis | 436 | **0.67(0.61-0.73)** | 15 | **4.32(2.61-7.17)** | 205 | **2.86(2.5-3.28)** | 144 | **0.61(0.52-0.72)** | 72 | **0.21(0.17-0.27)** |
| Aortic Aneurysm and Dissection | 282 | **0.6(0.53-0.67)** | 17 | **9.01(5.6-14.5)** | 101 | **2.42(1.99-2.94)** | 116 | **0.72(0.6-0.86)** | 48 | **0.18(0.13-0.24)** |
| Other Diseases of Arteries- Arterioles- Capillaries | 194 | **0.67(0.58-0.77)** | 6 | — | 63 | **2.57(2.01-3.29)** | 73 | **0.75(0.6-0.94)** | 52 | **0.31(0.24-0.41)** |
| Atherosclerosis | 159 | **0.61(0.52-0.71)** | 7 | — | 38 | **1.93(1.4-2.65)** | 67 | **0.79(0.63-1.01)** | 47 | **0.3(0.22-0.4)** |
| Other cancers (Non-prostate) causes of death | 2064 | **0.12(0.12-0.13)** | 176 | **2.45(2.12-2.84)** | 648 | **0.41(0.38-0.45)** | 796 | **0.14(0.13-0.15)** | 444 | **0.05(0.04-0.05)** |

Bolded SMRs are significantly different from 1.00 (P < .05). A blank indicates that because of 0 observed deaths, the SMR, 95% CI, and P value were not calculated
